# Development of Alive! (A Lifestyle Intervention Via Email), and Its Effect on Health-related Quality of Life, Presenteeism, and Other Behavioral Outcomes: Randomized Controlled Trial

**DOI:** 10.2196/jmir.1112

**Published:** 2008-11-19

**Authors:** Gladys Block, Barbara Sternfeld, Clifford H Block, Torin J Block, Jean Norris, Donald Hopkins, Charles P Quesenberry, Gail Husson, Heather Anne Clancy

**Affiliations:** ^2^Division of ResearchKaiser PermanenteOaklandCAUSA; ^1^NutritionQuestBerkeleyCAUSA

**Keywords:** Physical activity, diet, randomized controlled trial, evidence-based medicine, intervention studies, occupational health, community health services, employer health costs, health promotion, preventive health services

## Abstract

**Background:**

Cost-effective interventions to improve diet and physical activity are a public health priority. *Alive!* is an email-based intervention to increase physical activity, reduce saturated and trans fats and added sugars, and increase fruit and vegetable consumption. It was shown to improve these behaviors in a large randomized controlled trial.

**Objective:**

(1) To describe the components and behavioral principles underlying *Alive!*, and (2) to report effects of the intervention on the secondary outcomes: health-related quality of life, presenteeism, self-efficacy, and stage of change.

**Methods:**

The *Alive!* behavior change model is designed to elicit healthy behaviors and promote their maintenance. Behavioral strategies include assessments followed by individualized feedback, weekly goal-setting, individually tailored goals and tips, reminders, and promotion of social support. *Alive!* was tested among non-medical employees of Kaiser Permanente of Northern California, who were randomized to either the intervention group or the wait-list control group. After randomization, intervention group participants chose one topic to undertake for the intervention period: increasing physical activity, increasing fruits and vegetables, or decreasing saturated and trans fats and added sugars. Pre-post questionnaires assessed changes in SF-8 health-related quality of life, presenteeism, self-efficacy, and stage of change. Mixed effects multiple linear regression and ordinal logistic regression models were used, with department as a random effect factor. Analyses were by intention to treat: the 30% (238/787) who did not respond to the follow-up questionnaires were assigned change scores of zero.

**Results:**

Participants were 19 to 65 years (mean 44.0 +/- 10.6), and 74.3% (585/787) were female. Mean SF-8 Physical quality of life score increased significantly more in the intervention group than in the control group, 1.84 (95% CI 0.96-2.72) vs 0.72 (95% CI -0.15-1.58) respectively, *P* = .02. SF8 Mental score also improved significantly more in the intervention group than in the control group (*P* = .02). The odds ratio for improvement in self-assessed health status was 1.57 (95% CI 1.21-2.04, *P* < .001) for the intervention group compared to the control group. The odds ratio for having a reduction in difficulty accomplishing work tasks because of physical or emotional problems, a measure of presenteeism, was 1.47 (95% CI 1.05-2.05, *P* = .02) for the intervention group compared to the control group. The odds of having an improvement in self-efficacy for changing diet was 2.05 (95% CI 1.44-2.93) for the intervention vs the control group (*P* < .001). Greater improvement in stage of change for physical activity (*P* = .05), fats (*P* = .06), and fruits/vegetables (*P* = .006) was seen in the intervention group compared to the control group. Significant effects on diet and physical activity behavior change are reported elsewhere.

**Conclusions:**

Cost-effective methods that can reach large populations with science-based interventions are urgently needed. *Alive!* is a fully automated low-cost intervention shown to effect significant improvements in important health parameters.

**Trial Registration:**

Clinicaltrials.gov NCT00607009; http://clinicaltrials.gov/ct2/show/NCT00607009 (Archived by WebCite at http://www.webcitation.org/5cLpCWcT6)

## Introduction

The important role of diet and physical activity in reducing the burden of chronic disease and obesity is well-established [1]. Chronic diseases are responsible for 5 of the top 6 leading causes of death, as well as for decreases in quality of life. Much of the chronic disease burden is preventable [1]. Diets high in saturated and trans fats contribute substantially to coronary heart disease [2,3] and to cancers of the colon, breast, and prostate [4,5]. Low fruit and vegetable intake is associated with increased risk of 14 specific cancer types [4]. Physical inactivity is strongly associated with coronary heart disease [6,7], Type II diabetes [8,9], colon cancer, and possibly breast cancer [10,11]. Thus, improvements in dietary habits and physical activity can reduce the risk of obesity and of many chronic diseases.

Despite the substantial evidence linking these behaviors to health outcomes, the great majority of Americans do not meet dietary and physical activity guidelines [12-15]. More than half of US adults do not get enough physical activity to provide health benefits, including approximately one-fourth who are sedentary [1]. Similarly, only one-fourth of US adults consumes 5 or more fruits and vegetables per day [1].

Intervention programs can change these behaviors, and a number of on-site and face-to-face programs have been found to be effective, such as those described by Beresford et al [16] and Proper et al [17]. However, there is a large gap between the development of effective interventions and their extensive use in industry or public health practice [18,19]. While there are many barriers that impede translation of research into widespread practice, one significant obstacle has been the high cost and large time demands on both staff and participants [19]. As noted by Glasgow and Emmons [19], using lower cost intervention strategies, such as mail, phone, or computer-based approaches, may have the potential to overcome this limitation and make it possible to deliver effective behavior change interventions to large numbers of participants.

A number of research groups have developed effective mailed or computer-based and computer-tailored interventions, including Campbell et al [20], Gans et al [21], Marcus et al [22], and Brug et al [23]. Use of the Internet and email can greatly extend the reach of such programs. Significant improvements in diet and/or physical activity behaviors through use of Internet-based strategies compared with no-intervention controls have been shown by Oenema et al [24], Spittaels et al [25], Napolitano et al [26], Hurling et al [27] and others. Other interventions for physical activity have been reviewed by van den Berg et al [28]. Effective Internet-based programs to promote or maintain weight loss have also been developed [29,30]. The improvements in health and productivity resulting from some of these programs have even been shown to reduce employer costs [31].

*Alive!* (A Lifestyle Intervention Via Email) is an email-delivered, computer-tailored program to reach individuals on a large scale with an intervention which applies effective behavior-change principles. It is a modification of a previous program, WIN (Worksite Internet Nutrition), a computer-tailored, email-delivered program which was tested at a worksite and found to be effective in a pre-post analysis [32]. *Alive!* was developed in a collaboration between the Kaiser Permanente of Northern California Division of Research and NutritionQuest (formerly known as Block Dietary Data Systems). It is designed to achieve behavior change in physical activity and diet. In the dietary component, the targets are increases in fruits and vegetables and decreases in saturated and trans fats and added sugars. The development of *Alive!* and subsequent trial were funded by the Centers for Disease Control and Prevention (CDC) as part of the Health Protection Research Initiative emphasis on Worksite Health Promotion, which focused on interventions at worksites.

The primary outcomes of the randomized controlled trial were change in diet and physical activity. Those results are in preparation [33] and are summarized below. The decision to report the primary results of this trial in a different paper in a different journal was made because the content of the two papers was different, and because we wished to communicate our primary diet and physical activity behavior change results broadly to persons engaged in health promotion and preventive medicine. The trial was conducted among regional non-medical employees of Kaiser Permanente of Northern California. In comparison with change in the control group, the intervention group showed significant increases in minutes per week of moderate intensity activity, vigorous intensity activity, and walking; significant increases in fruits and vegetables; and significant decreases in saturated fat and trans fats. Decreases in added sugars in comparison with change in the control group approached statistical significance.

Here we describe the components and principles of the *Alive!* program, and report results of secondary outcomes of the *Alive!* trial, including health-related quality of life, self-assessed health status, presenteeism, self-efficacy, and stage of change. These are important outcomes in themselves, and the effect of presenteeism on productivity in particular is important in increasing the usage of wellness programs among employers.

## Methods

### Overview of Alive!

*Alive!* is designed to assist individuals in increasing their physical activity, increasing their fruit and vegetable intake, and decreasing their intake of saturated and trans fats and added sugars. *Alive!* is not a weight loss program; the focus is entirely on improving these nutritional and physical activity health behaviors. It is a completely automated system, in which all the content and tailoring is contained in the computerized program, and is delivered entirely via email. No additional professional or technical expertise is required for the delivery. Potential participants may be invited to try *Alive!* through a batch email sent by the leaders of a business or organization to its employees or members. Completion of the initial step, health risk assessments on diet and physical activity, is encouraged by promising immediate feedback on their levels of those behaviors, regardless of whether or not they decide to participate further in *Alive!*. If they do decide to participate in the full program, participants choose an initial health-behavior module to work on for the subsequent 3 months, either to: (1) increase physical activity, (2) increase fruits and vegetables, or (3) decrease saturated and trans fats and added sugars. Participants then receive weekly messages offering tailored small-step goals to choose for the following week, tailored tips for achieving those goals, health information, and numerous opportunities for interaction and engagement. Information exchanged between client and server is encrypted by the industry standard security protocol, Secure Sockets Layer. Midweek messages remind the participants of the small-step goals they chose to work on for the week. A total of 25 personalized program-initiated email contacts occur over a single 3-month intervention period. In a non-research setting, participants may re-enroll in subsequent 3-month intervention periods, potentially covering all three topics over one year. The use of *Alive!* in the Kaiser trial differed slightly from the standard *Alive!* program, in that participants chose only a single topic, and the intervention lasted for a single 4-month period rather than 3 months, with messages sent weekly for the first 2 months and then every other week for the final 2 months.

### Features of the Alive! Program

#### Baseline Assessments and Feedback

Diet and physical activity health risk assessments (HRAs), described in more detail below, are delivered via email and take approximately 15 minutes to complete.

##### Physical Activity

The physical activity questionnaire was adapted from the Cross-Cultural Activity Participation Study (CAPS) questionnaire [34]. It contains 34 specific activities, divided into domains that include walking, biking and other transportation, caregiving and household chores, conditioning exercises, dance and sports, and other leisure activities, such as watching TV or videos. Respondents are asked to indicate how many days a week and how many minutes a day they participate in each of the activities in a typical week in the past 4 months. Each activity is assigned a MET value (a measure of energy expenditure where 1 MET is equivalent to the energy required for sitting quietly) according to the Compendium of Physical Activities [35], multiplied by frequency and duration, and then summed over all relevant activities to create the summary variables. Five physical activity variables are estimated: total activity, in MET-minutes/week; moderate intensity and vigorous intensity physical activity, walking, and sedentary behavior, all in minutes/week. Four-month test-retest Spearman reliability (reproducibility) coefficient for minutes of moderate activity among the control group in the *Alive!* trial was 0.67.

##### Diet

The dietary questionnaire contains 35 items, asks about “usual” intake, and includes both frequency and portion size. Foods were identified for inclusion based on analyses of the National Health and Nutrition Examination Survey (NHANES) 1999-2004 [36], with separate analyses for African Americans, Whites, and Hispanics to ensure inclusion of foods appropriate for those ethnic groups. Foods were included if they were important contributors of saturated fat, trans fats, fruits and vegetables, or added sugars. Nutrient content was based on the US Department of Agriculture’s Food and Nutrient Database for Dietary Studies [37] as well as on published data and label values. Nutrient estimates are calculated by multiplying frequency, portion size, and nutrient content and summing over all foods [38]. Additional questions on types of food consumed (eg, type of milk) permit more precise estimates of saturated and trans fats and sugars. The trans fat values are based only on hydrogenated products, and do not include trans fats from animal products. The database was developed and the randomized trial was conducted after the US Food and Drug Administration labeling regulations for trans fats went into effect [39]. Four-month test-retest reliability (reproducibility) of the dietary questionnaire ranged from 0.70 to 0.78, indicating good reliability. The questionnaire is a variant of widely-used Block questionnaires.

##### Tailoring/Lifestyle Questionnaire

A second questionnaire, again delivered via email, obtains demographic data, tailoring information, and information related to assessing secondary outcomes. Tailoring information includes presence of children at home, habits related to cooking and eating out, physical activity preferences such as structured, facility-based exercise or lifestyle physical activity, and stage of readiness for change [40] for physical activity. In addition, extensive tailoring is also based on specific foods and activities reported in the diet and physical activity questionnaires.

##### Barriers Questionnaire

In this questionnaire, participants identify barriers that may get in the way of achieving their health behavior goals. Subsequent messages provide tips for overcoming their reported barriers.

##### Feedback From the HRA

Feedback is provided immediately after the participant submits the HRA. Separate reports are made of the participant’s intake of saturated fat, trans fats, added sugars, fruits and vegetables, and amount of physical activity, in relation to national and international guidelines [41-45]. Where improvement is needed, the feedback provides brief suggestions, including information on the participant’s top three sources of problematic nutrients, and of sedentary behavior. This feedback also provides the participant with a basis on which to choose the health behavior to work on in the coming months. See Multimedia Appendix 1 for examples of the assessment and feedback.

After receiving the feedback, individuals may choose to participate in the full *Alive!* program. At that point they choose the overall health behavior objective to work on: Physical Activity, Fats and Sugars, or Fruits and vegetables.

#### Tailored Goal-setting

##### Tailored Goal-setting Is the Core of the Alive! Program

**Figure 1:**
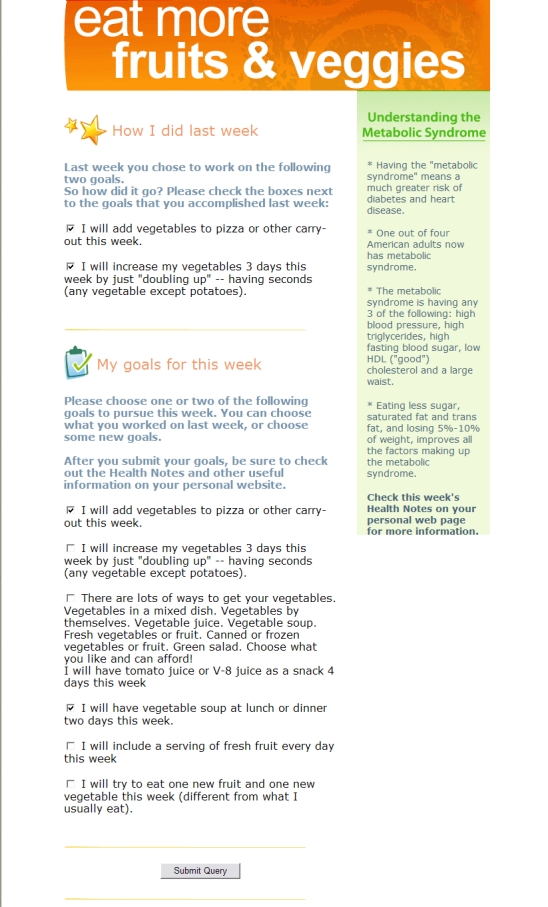
Example of weekly email

Each week, the participants receive an email suggesting four to six small-step goals which are tailored to the individual characteristics mentioned above (Figure 1).

Participants are asked to commit to one or two of these to work on for the following week. The purpose of the tailoring is to identify small-step goals that are relevant to the individual participant and that take into account his or her constraints and preferences. These are small achievable goals, such as “I will have a salad with lunch two days this week” or “I will walk 20 minutes at lunch time”. Dietary goals are also suggested based on the individual’s reported intake. For example, a person who eats doughnuts twice a week may receive a suggested goal to eat them only once a week, or to eat a smaller portion. Physical activity goals are also tailored to a combination of stage of change and initial level of activity: persons reporting precontemplation or low/no activity will initially be given goals that facilitate their getting started, such as easy walking or buying walking shoes. Table 1 contains examples of tailoring characteristics and associated goals.

In subsequent weeks, in the email delivering the next set of goals, the participant is also asked whether or not the previous week’s goals were achieved. This is recorded in the Goal Tracker (see below).

**Table 1:** Illustrative tailoring characteristics and associated suggested goals

Characteristic of Participant	Sample Small-Step Goal
**Physical Activity Path**	
Early stage, prefers lifestyle activities, no children at home	I will make a date with a friend to go for a walk instead of for coffee or a drink.
Early stage, has children at home	I will go to the playground with my kids two days this week after school/work, and walk around the playground.
Action stage, prefers exercise activities, has children at home	I will get a family fitness video or DVD and do it with my kids at least one day this week.
Action stage, prefers lifestyle activities, no children at home	I will walk to do errands or window shop on my lunch hour rather than sitting in cafeteria or at my desk, at least two days this week.
**Fats/Carbs Path**	
Most dinners eaten at home, participant does the cooking	This week I will buy olive oil, and use it when I fry or stir-fry
Eats out frequently	I will look for opportunities to eat whole grain foods when I eat out this week.
Conditional (eats sweetened cereal)	This week when I shop, I will read the label on the box, and choose a cereal with less sugar.
Conditional (eats sweetened cereal) and has children at home	This week when I shop, I will show my children how to read the label, and choose a cereal with less sugar.
**Fruits/Vegetables Path**	
Eats out frequently	I will add vegetables to pizza or other carry-out this week.
Most dinners eaten at home, no children at home	I will try to eat one new fruit and one new vegetable this week (different from what I usually eat).
Most dinners eaten at home, children at home	I will have the kids participate in grocery shopping this week and choose one vegetable or fruit they are willing to eat.
Participant does the cooking	On two days this week, I will build vegetables into the main dish, like adding frozen green beans to stew.

##### Mid-week Reminders

A brief email mid-week reminds the participants of the goals they have chosen.

#### User’s Home Page

Immediately after choosing a goal, the participant is taken to his or her “personal home page” containing tips for achieving the goal(s) they have chosen, tips regarding the barriers they mentioned, a goal tracker, an interactive simulation tool, health information, and links to sites for additional information, such as government and organizational websites. Thus, the act of choosing a goal in the email reader ensures that 100% of participants who choose a small-step goal for the week will also view the additional home page content described below; no additional initiative on the part of the participant is required. See Multimedia Appendix 2 for example of weekly email and home page.

##### Tips

Each week, participants receive tips on ways to achieve the specific small-step goals they have chosen that week; tips are also tailored to the factors above. They also receive tips on how to handle specific barriers that the participant has reported as constraints, such as time, money, or travel.

##### Goal-tracker

The program tracks which goals the participant has successfully achieved and categorizes them as to type of goal (eg, change in frequency vs change in amount). This is available on the participant’s personal home page. This information was not used in the evaluation of the effectiveness of the program, but was provided as an aid to the participant in understanding what types of goals work for that individual.

##### Simulation Tool

The simulation tool is an interactive feature of the *Alive!* program that allows participants to see a graphic presentation of how much any specific change in diet or physical activity might move them closer toward the recommended level. The tool is linked to the participant’s responses to the diet and physical activity questionnaires, and “remembers” both the individual’s baseline score (eg, for saturated fat), the recommended level for that score, and the participant’s baseline responses to each question. With it, the participant can experiment with changing aspects of his or her diet or physical activity and see a visual representation of how much such a change might move him or her toward the recommended level. Any of the 35 foods can be manipulated in terms of their frequency, portion size, or type. The 34 physical activity behaviors can be manipulated in terms of frequency and duration. For example, people who drink whole milk could change either the frequency, the portion size, or the type and see how much closer they would be to the saturated fat goal. Similarly, people who walk once a week for 15 minutes could see how much closer they would be to the physical activity recommendation if they walked three times a week for 20 minutes, and so forth.

##### Health Information

Each week, a different topic relevant to the selected intervention objective (ie, physical activity, fruits/vegetables, or carbs/fats) is discussed in a “Health Note”. Topics include research on the relation of physical activity, fruits and vegetables, or saturated and trans fats to heart disease, healthy weight, various cancers, metabolic syndrome, mental health, and cognitive decline. Knowledge relevant to the particular intervention objective is also provided, such as the components of fitness, trends in physical activity, and different types of fats. A brief summary of the topic appears in each weekly email, and the full article is presented on the individual’s personal home page.

##### Provisions for Social Support

Weekly suggested goals and tips promote building social support by suggestions such as walks with colleagues at lunch time. Equally important, *Alive!* encourages participants to invite family members to join *Alive!* to increase social support for behavior change. Finally, a chat room provides an opportunity for participants to discuss problems with each other and suggest solutions.

### Principles Underlying the Alive! Program

The behavioral strategies underlying *Alive!* include certain of the principles from the health belief model [46], the theory of reasoned action [47], social cognitive/social learning theories [48], goal-setting theory [49], social marketing [50], and the transtheoretical model [51], all derived from behavioral and cognitive psychology. All of these theories suggest various concrete behavioral management strategies, such as setting goals, self-monitoring, anticipating barriers, rewarding accomplishments, and increasing knowledge and skills, as ways to elicit and reinforce the desired behavior. *Alive!* was not designed to test any particular model, but rather it incorporates elements from these various models which have been proven to be important in initiating and sustaining behavior change.

### The Alive! Behavior Change Model

**Figure 2:**
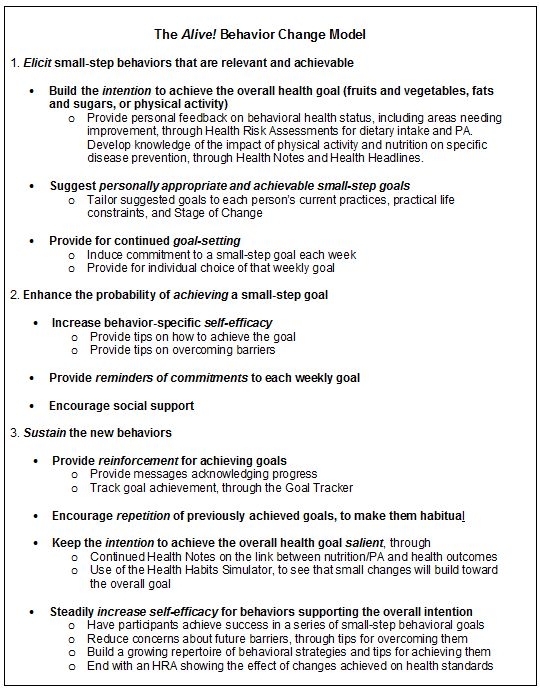
The Alive! behavior change model

These behavioral strategies are applied in a basic structure of bringing forth a desirable behavior and providing the cues and repetition that help make the new behaviors habitual (Figure 2). Initially, *Alive!* promotes or reinforces the intention to change behaviors. It then moves to elicit specific behaviors by requesting commitment to small achievable weekly goals. It helps in achieving that commitment through tips and reminders, and it promotes sustaining the new behaviors through a variety of means as shown in Figure 2.

### The Randomized Controlled Trial

#### Study Design and Sample

**Figure 3:**
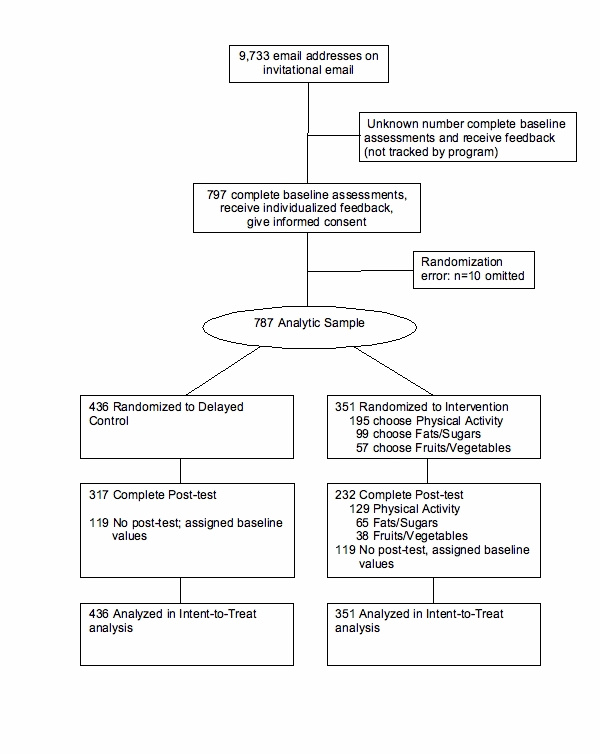
Randomized controlled trial intervention and follow-up

A randomized controlled trial was conducted among non-medical regional employees of Kaiser Permanente of Northern California (KP). Persons employed in the Kaiser Division of Research, of which Dr. Sternfeld is a member, were not eligible to participate. Recruitment began in July 2006 and was accomplished in approximately three weeks. The intervention and follow-up was completed in December 2006 (Figure 3). Procedures were approved by the Northern California Kaiser Permanente Institutional Review Board. The primary objective was to test the effectiveness of *Alive!* in changing diet and physical activity. Those results will be reported elsewhere. Target sample size was based on the number and size of departments and 80% power to detect a difference in mean change scores in diet and physical activity across a reasonable range of probable intraclass correlations between baseline and follow-up. The primary hypotheses tested in the trial were that participation in *Alive!* would produce significantly greater improvement in physical activity and the targeted dietary behaviors in the intervention group in contrast with the control group. The prespecified secondary hypotheses were that participation in *Alive!* would produce significantly greater improvement in quality of life and presenteeism in the intervention group in contrast with the control group. We additionally examined treatment effects on stage of change and self-efficacy.

Employees were recruited through an invitational email sent from KP administrative offices, which included the diet and physical activity questionnaires described above. All employees were eligible. There was no monetary incentive to participate in the assessment or the subsequent randomized trial. The number who took the assessments and received individualized feedback but did not choose to join the randomized trial was not tracked.

Persons who agreed to participate in the randomized trial were automatically randomized by the program to either the intervention group or the control group. Randomization was by department (n = 192 departments) after stratification by department size, using a random number table. The control group was a delayed control, and control group participants were offered the program 8 months after the initial randomization. Thus, participants were aware of their randomization group. The delivery of the intervention was completely automated and did not involve any investigator actions. Diet and physical activity questionnaires and questionnaires on secondary outcomes like health status were automatically administered by the program at baseline and at the conclusion of the intervention.

After randomization, participants in the intervention group chose the intervention path they wanted to pursue: Physical Activity (PA); Fruits and Vegetables (FV); Fats and Sugars (FS). Participants received intervention messages only for the chosen path. Neither participation in *Alive!* nor choice of an intervention topic was limited to persons with poor dietary or physical activity behaviors, and a substantial proportion had diet and physical activity behaviors within the recommended range at baseline.

#### Assessment of Study Outcomes

Data for secondary outcomes include self-assessed health status and health-related quality of life, using the SF-8 Health Survey questions [52]; presenteeism [53,54]; Stage of Change [40]; and self-efficacy [55] in physical activity and each of the dietary behaviors. Results were assessed by emailed questionnaire at baseline and after the 4-month intervention, administered automatically by the program.

The SF-8 Health Survey [52] is a set of quality-of-life measures, consisting of eight questions, representing eight domains of physical and mental health. The items are scored on a 5-point Likert scale, with the exception of self-assessed health status, which is scored on a 6-point scale from Excellent to Very Poor. Results were analyzed using the scoring algorithm provided for the instrument. This produces a standardized scoring permitting comparison with national data [56,57].

Presenteeism [53,54] is a concept that refers to reduced worker productivity resulting from mental and physical conditions, despite being present on the job, and has been shown to be a major contributor to the health-related costs of employers [58]. Presenteeism was assessed with three questions. Two questions asked about the number of hours in a typical 8-hour day that back pain or depression/anxiety interfered with accomplishing tasks at work. Response was provided in number of hours (0-8) and results were scored as decreased vs increased or stayed the same. The third was a question patterned after the SF-8 questions: “During the past 4 weeks, how much difficulty did you have concentrating at work and accomplishing work tasks because of physical or emotional problems?” The response pattern was a five-point scale ranging from “Not at all” to “Could not do my job work”.

Self-efficacy [55] was assessed with two questions: “How confident are you that you can make changes to be more physically active?” and “How confident are you that you can make changes to eat more fruits and vegetables and to reduce sweets, trans fat and saturated fat?”. In both cases the response categories were “Not at all”, “Somewhat”, and “Very confident”.

Stage of Readiness for Change [40] was assessed separately for change in fats, added sugar, fruits and vegetables, and physical activity.

#### Data Analyses

Results were analyzed by strict intention to treat, in which persons who did not respond to the follow-up questionnaire and therefore had missing data are included in the intention-to-treat analysis and assigned a change score of zero (119 of 436 in the control group, 27.3%; and 119 of 351 in the intervention group, 33.9%). Ordinal logistic regression models (Proc Genmod, SAS Institute, Cary, NC) were used for analyses of ordinal change variables. Multiple linear regression models (Proc Mixed, SAS Institute, Cary, NC) were used for analyses of the change in SF-8 quality of life. In all models, change in behavior was the dependent variable, randomization group was the primary fixed effect, department was a random effect factor, and all models were adjusted for age, gender, ethnicity, and baseline value of the dependent variable. Results are presented for the overall comparison of the intervention and control groups, although it should be noted that participants only received messages and goals relevant to the specific chosen path (Physical Activity, Fruits/vegetables, Fats/Sugars). Results presented as intention-to-treat probably represent an underestimate of effects, since they include all randomized participants including non-responders to the follow-up questionnaire (these were deemed to have a change score of zero, even though some of the non-responders may have experienced improvements in these behaviors).

## Results

### Participants

The trial includes 787 persons who gave informed consent to be randomized. A larger number completed the assessments and received feedback, but that number is not tracked by the system. Of the 787 participants in the trial, 351 (45%) were randomized to the intervention group and 436 (55%) to the control group. The mean age was 44 years (range 19-65 years), 202 (25.7%) were men, and 70% had a college degree or higher education (Table 2). The post-test questionnaire at the end of the 4-month intervention period was completed by 70.0% (549 of 787). Responders and non-responders to the post-test did not differ significantly in gender, education, or BMI category, but were significantly older (mean 44.8 vs 42.3 years) (data not shown).

**Table 2:** Demographic characteristics by treatment group and by intervention path (Alive! randomized trial, Oakland, CA 2006)

	Treatment Group			Intervention Path			
	Intervention	Control	*P*^a^	PA	Fruits/vegs	Fats/carbs	*P*^b^
**n (%)**	351 (45.0)	436 (55.0)	—	195 (55.6)	57 (16.2)	99 (28.2)	—
**Age (yrs), mean (sd)**	44.8 (10.0)	43.5 (11.0)	.09	45.3 (0.71)	42.7 (1.32)	44.9 (1.00)	.22
**Age category, n (%)**			.09				.05
< 35	63 (18.0)	106 (24.3)		32 (16.4)	10 (17.5)	21 (21.2)	
35-50	173 (49.3)	195 (44.7)		94 (48.2)	37 (64.9)	42 (42.4)	
> 50	115 (32.8)	135 (31.0)		69 (35.4)	10 (17.5)	36 (36.4)	
**Gender, n (%)**			.42				.16
Women	256 (72.9)	329 (75.5)		148 (75.9)	43 (75.4)	65 (65.7)	
Men	95 (27.1)	107 (24.5)		47 (24.1)	14 (24.6)	34 (34.3)	
**Ethnicity, n (%)**			.005				.80
African American	25 (7.1)	33 (7.6)		12 (6.2)	4 (7.0)	9 (9.1)	
Asian	28 (8.0)	39 (8.9)		19 (9.7)	3 (5.3)	6 (6.1)	
Latino	14 (4.0)	18 (4.3)		6 (3.1)	3 (5.3)	5 (5.1)	
White	111 (31.6)	188 (43.1)		62 (31.8)	21 (36.8)	28 (28.3)	
Mixed/Unknown	173 (49.3)	158 (47.7)		96 (49.2)	26 (45.6)	51 (51.5)	
**Education, n (%)**			.43				.29
High school or less/Some college	97 (27.6)	138 (31.7)		61 (31.3)	12 (21.1)	24 (24.2)	
College grad	119 (33.9)	145 (33.3)		59 (30.3)	20 (35.1)	40 (40.4)	
Graduate/ professional degree	135 (38.5)	153 (35.1)		75 (38.5)	25 (43.9)	35 (35.4)	
**Children living at home, n (%)**			.85				.78
Yes	153 (43.6)	193 (44.3)		88 (45.1)	23 (40.4)	42 (42.4)	
No	198 (56.4)	243 (55.7)		107 (54.9)	34 (59.7)	57 (57.6)	
**Body mass index,** **mean (sd)**	28.5 (6.8)	28.7 (7.5)	.74	30.0 (0.47)	25.7 (0.87)	27.3 (0.66)	< .001
**BMI category, n (%)**			.30				< .001
< 25	123 (35.0)	165 (37.8)		56 (28.7)	29 (50.9)	38 (38.4)	
25-29.9	117 (33.3)	123 (28.2)		59 (30.3)	21 (36.8)	37 (37.4)	
30-34.9	55 (15.7)	63 (14.5)		35 (18.0)	5 (8.8)	15 (15.2)	
35 and above	56 (16.0)	85 (19.5)		45 (23.1)	2 (3.5)	9 (9.1)	

^a^ *P* values from *t* test for difference in means or chi-square test for differences in proportions between intervention and control groups.

^b^ *P* values from ANOVA for differences among intervention paths.

### Health-related Quality of Life (SF-8)

At baseline, the mean and standard deviation (SD) was 49.9 (7.9) and 48.0 (9.6) for the SF-8 Physical and SF-8 Mental summary scores respectively. The effect of treatment was significant for the two summary variables: change in these factors was significantly greater in the intervention group compared to the control group (*P* = .02) (Table 3). There was a significantly greater likelihood of having improvement in self-assessed health status in the intervention group vs the control (OR=1.57, 95% CI 1.21-2.04, *P* < .001). Several other components of the SF-8 were significant, including Role Physical, Bodily Pain, and Mental Health (data not shown).

**Table 3:** Effect of Alive! on SF-8 summary measures and self-assessed health status: change in the intervention group vs change in the control group

	Adjusted Mean Change (MC)^a^ or Odds Ratio (OR)^b^ (95% Confidence Interval)^a^		*P*
Variable	Intervention	Control	
SF-8 Physical^a^	MC 1.84 (0.96 -2.72)	MC 0.72 (-0.15 - +1.58)	.02
SF-8 Mental^a^	MC 0.69 (-0.28 - +1.67)	MC -0.29 (-1.22 - +0.65)	.02
Self-Assessed Health Status (SF8 “General health”)^b^	OR 1.57 (1.21 - 2.04)		< .001

^a^Adjusted mean change and significance from mixed models with department as random effect factor and adjusted for baseline value, age, sex, and ethnicity. *P*-value represents significance of the difference between change in the intervention group and change in the control group. Intention-to-treat models, non-responders set to zero change.

^b^Odds ratio and significance, odds of having a reported improvement in general health, in the intervention group vs the control group. Model from ordinal logistic regression, with randomization group as primary fixed effect, department as random effect factor and adjusted for baseline value, age, sex, and ethnicity.

### Presenteeism

The proportion of the sample reporting greater than zero hours for difficulty concentrating and accomplishing work tasks because of back pain or depression/anxiety at baseline was 22.5% (177/787) and 30.6% (241/787) respectively (data not shown). Decrease in number of hours of back pain and depression in the intervention group vs the control group approached significance, while differences in the third presenteeism measure were significant (Table 4). Persons in the intervention group were 1.47 times more likely to report improvement in the ability to concentrate and accomplish work tasks (*P* = .02) in comparison with changes in the control group.

**Table 4:** Effect of Alive! on presenteeism^a^: change in the intervention group vs change in the control group

Variable	Odds Ratio (95% CI)	*P*
Decreased hours of back pain at work^b^	1.66 (0.99 - 2.79)	.054
Decreased hours of depression at work^b^	1.74 (0.98 - 3.10)	.06
Change in Concentrate/accomplish^c^	1.47 (1.05 - 2.05)	.02

^a^Presenteeism refers to the situation in which the employee is present at work, but productivity is reduced as a result of physical or mental conditions. Intention to treat models, everyone included, non-responders set to zero change. Models from dichotomous or ordinal logistic regression with department as random effect factor and adjusted for baseline value, age, sex, and ethnicity.

^b^Odds ratio and significance, odds of having a decrease in pain or depression, in the intervention group vs the control group. Questions were asked in following format: “During a typical 8-hour workday, about how many hours does BACK PAIN interfere with concentrating on work and accomplishing work tasks?”. Range of responses was 0-8. Change scored as 1 = hours decreased, 0 = hours stayed the same or increased.

^c^Odds of having improvement, intervention group vs. control group. Ordinal logistic regression with department as random effect factor, and adjusted for baseline. Question was asked in following format: “During the past 4 weeks, how much difficulty did you have concentrating at work and accomplishing work tasks because of physical or emotional problems?”

In Table 5 below, we examine change in efficacy for diet and physical activity in the entire intervention group, and we evaluate change in stage separately for fats, sugars, fruits/vegetables, and physical activity in the entire intervention group. However, it should be noted that participants in the intervention group received goals and interactions with regard to only one of the three intervention topics (physical activity, fruits/vegetables, or carbs/fats).

### Self-efficacy

Persons in the intervention group had significantly greater improvement in confidence in ability to change their diet than did those in the control group (Table 5). For physical activity, confidence did not improve significantly in the intervention group compared to the control group, when all subjects are examined, including those “Very Confident” at baseline. However, it is notable that even there the direction of the effect is positive (odds ratio > 1.0), despite the fact that the only direction possible for those already “Very confident” was either no change or decrease. When change in confidence to improve physical activity is examined just in those in the Physical Activity path who were not already “Very confident”, a significant improvement is seen (*P* = .037) (data not shown).

### Stage of Readiness for Change

When all subjects are included, including those in Maintenance at baseline and thus with no room to improve, there was significant or almost significant forward movement in Stage in the intervention group in comparison with change in the control group for all domains except for change in sugar (Table 5). Among those needing improvement (“at risk”), significant forward movement was seen in all domains. The substantially greater effect on Stage of Change for sugar in the at-risk group is evidence of the large number of participants who were already in Maintenance for reducing sugar intake at baseline. When only those in the relevant path are examined (eg, movement in Stage for physical activity among those in the Physical Activity path), there was significant movement in all domains (data not shown).

**Table 5:** Effect of Alive! on self-efficacy and stage of readiness for change: change in the intervention group vs change in the control group

	Intention-to-treat		Intention-to-treat	
	At-risk subjects^a^		All subjects^a^	
	Odds Ratio (95% CI)^b^	*P*^c^	Odds Ratio (95% CI)^b^	*P*^c^
**Self-efficacy analyses**				
Self-efficacy to change diet	2.68 (1.57 - 4.57)	< .001	2.05 (1.44 - 2.93)	< .001
Self-efficacy to change physical activity	1.42 (0.98 - 2.07)	.07	1.21 (0.87 - 1.67)	.26
**Stage-of-change analyses**				
Stage: Changing fat	1.32 (1.00 - 1.76)	.05	1.27 (0.99 - 1.63)	.06
Stage: Changing fruits/vegetables	1.76 (1.31 - 2.36)	< .001	1.62 (1.23 - 2.13)	.006
Stage: Changing added sugars	1.84 (1.31 - 2.58)	< .001	1.23 (0.92 - 1.64)	.17
Stage: Changing physical activity	1.42 (1.06 - 1.90)	.02	1.34 (1.00 - 1.80)	.05

^a^In intention-to-treat models, subjects who did not respond to the follow-up questionnaire have their change score set to zero. CI: 95% Confidence Interval. “All Subjects”: Subjects in Maintenance (for Stage analysis) or “Very confident” (for Self-efficacy analysis) at baseline are included. “At-risk Subjects”: Excludes those in Maintenance (or “Very confident”) at baseline.

^b^Odds ratio: Odds of having forward movement, intervention group vs control group.

^c^Significance of odds ratio for forward movement for intervention group vs control group from ordinal logistic regression models with department as random effect factor, adjusted for baseline value, age, sex, and ethnicity.

### Process and Satisfaction

The personalized report on their diet and physical activity behaviors, which was provided to all 787 participants prior to randomization immediately after completion of the baseline questionnaires, appears to have benefited those subsequently randomized to the control group as well as those randomized to the intervention group. Of control group respondents to the follow-up questionnaires at the end of the 4-month period, 89.1% (271/304) reported they learned “Some” or “A lot” about their physical activity behaviors, and 88.5% (269/304) reported they had learned “Some” or “A lot” about their dietary behaviors (data not shown). Results were similar for the intervention group. Among members of the intervention group, 154 of 224 respondents to the follow-up questionnaires (68.8%) found the tailored tips “Somewhat” or “Very” relevant and helpful. The chat room was infrequently used. However, participation in the key element of the *Alive!* program, goal-setting, was high: 74% of those randomized to the intervention group (260/351) interacted with the program on 7 or more of the 12 weeks, as tracked automatically by the program. In addition, the program automatically tracks goals selected by each participant. The 351 participants in the intervention group selected 3836 goals over the 3-month intervention period, or an average of 10.9 goals per person.

## Discussion

### Principal Results

*Alive!* was developed to provide a low-cost intervention capable of reaching large numbers of people with an intervention grounded in established principles of behavior change. These analyses demonstrate that the *Alive!* program promoted significant improvements in SF-8 health-related quality of life, presenteeism, self-efficacy, and stage of change. The significant improvements in diet and physical activity will be reported elsewhere.

#### Quality of Life

The effects on SF-8 measures and self-reported general health suggest a potentially important beneficial effect of participation in the *Alive!* program on the population’s physical and mental health and quality of life. The SF-8 instrument used here is a reduced version of the SF-36, measuring the same eight constructs [56], which has been extensively validated [57]. *Alive!* produced significant improvements for the overall SF8-Physical and Mental scores, even in intention-to-treat analyses where non-responders are set to zero change. The single-item, self-assessed health status question has been shown to predict mortality among middle-aged and older persons, even after control for health, demographic, and social factors [59-61], and has been suggested to be even more reliable than biomedical measures [62]. Other researchers have found beneficial effects on related variables as a result of Web or email-based interventions. Christensen et al [63] and Clarke et al [64] found significant effects of a depression-oriented Web-based intervention. The only researchers of which we are aware to have found significant improvement in a depression score as a result of an Internet-based program to improve diet and physical activity, like *Alive!*, are Kerr et al [65].

#### Presenteeism

A recent large study demonstrated that health-related productivity losses cost employers more than four times as much as medical and pharmacy costs [58]. Measures of presenteeism have been used in numerous studies that demonstrate the cost of such lost productivity [31,53,54]. Improvements in these sources of costs are of major interest to employers. Numerous studies have shown beneficial effects on absenteeism and presenteeism as a result of diet and physical activity interventions [31]. Our results suggest that *Alive!* can make a contribution to such improvements.

#### Self-efficacy

The improvements in self-efficacy shown here may have important implications for the longer-term impact of participation in *Alive!*, if they maximize the likelihood of sustaining the improved behaviors.

We believe that the demonstrated success of *Alive!* in achieving improvements in health-related quality of life, presenteeism, stage of change, and self efficacy, as well as diet and physical activity outcomes shown elsewhere, may be due, in part, to the nature of the tailoring variables. Rather than tailoring solely on psychosocial characteristics such as stage of change and self-efficacy, *Alive!*’s tailoring focused primarily on each individual’s current dietary and PA practices, and on their practical life constraints, with small-step goals and tips that took such habits, constraints, and barriers into account.

The approach of *Alive!*, and in its predecessor, WIN, is consistent with the concept of “Stickiness” [66]. The “stickiness” concept suggests that ideas and intentions are likely to “stick” when they are particularly relevant to an individual and when they appear frequently in the mental or social environment. *Alive!* is designed to increase relevance and stickiness in numerous ways. These include the feedback from the diet and physical activity questionnaires; tailored goals and tips; the Health Notes, which may strike a chord in some people and increase relevance; and repeated reminders. Reminders not only increase continued commitment but also enhance the salience of other cues in the environment such as news reports. The 25 contacts over 3 months, all on aspects of the same overall behavior, both reinforce the overall behavior and provide repeated opportunities for the “motivational storm” that can generate deep and sustained change.

### Reach and Engagement

In this study, the exact rate of participation in the randomized trial is not known, as there was no way to know how many of the 9733 email addresses were live nor how many of the invitational messages may have been spam-filtered. Our estimate is a participation rate of approximately 10%. This participation rate in the trial is reasonably consistent with other randomized trial experiences. As noted above, substantially more than 787 completed the assessments and received the feedback but did not choose to participate in the randomized trial. It is notable that there was no monetary incentive, and potential participants were told that they might not receive the intervention for 8 months if they were randomized to the control group. In addition, the participation rate was considerably higher than has been seen in some other Internet-based interventions. For example, Glasgow et al [67] found only a 2.4% participation rate among general non-diseased membership in an HMO, after a mailed letter of invitation.

Engagement in this intervention was substantial, with an average of 10.9 goals selected per person over the 12 intervention sessions, and with 74% of intervention group subjects interacting with the program on 7 or more of the 12 intervention sessions. This appears to be a substantially higher engagement than some researchers have seen in Internet-based programs for the general population. Glasgow et al [67] found that only 49% of the sample viewed at least one follow-up newsletter after the initial intervention message. Verheijden et al [68] found that only 9.6% used their Web-based health promotion site more than once.

### Limitations

Some limitations of *Alive!* should be noted. The requirement for email and Internet access limits the applicability of *Alive!* to some segments of the population. However, as of 2006, 73% of American adults were Internet users, including 71% of persons 50-64 years of age [69]. While fewer low-income people have Internet access, 53% of adults living in households with less than $30,000 annual income go online, as of 2006 [69].

It is also acknowledged that effect sizes are small in this intention-to-treat analysis in which those with missing data are assigned change scores of zero. It is worth noting that the trial randomized subjects even if they had already met diet or physical activity goals or were already at the top of scales such as efficacy and stage. Thus, the study differs fundamentally from classic “clinical” trials in which only at-risk subjects are randomized. It is also worth noting that participants chose a goal only after being randomized to the intervention or control groups, and thus each person in the intervention group participated in only one of the three intervention topics. Consequently, the generalized effects on efficacy, stage, and quality of life suggest a generalized halo effect on healthy behaviors and characteristics beyond the direct topic in which they participated.

Another limitation is the fact that there are no objective measures of outcomes like self-efficacy, quality of life, sick days, or productivity. Potential conflict of interest of some of the authors may also be noted as a limitation, as NutritionQuest developed *Alive!* and has proprietary interests in it. However, the principal investigator of the randomized trial (BS) has no financial interest in *Alive!*, and all statistical analyses were either performed or confirmed by Kaiser statistical staff.

A notable strength of *Alive!* is its ability to reach very large numbers of people with a fully automated, quite intensive intervention grounded in effective behavior change principles. Marcus et al [70] note that “evidence supports individually tailored behavior-change-oriented programs at the workplace”. Marcus et al also note that a major limitation of many studies is their failure to incorporate cognitive principles. In addition, many successful programs, although grounded in theory, fail to be effectively translated to the “real world” because they place too great a burden on organization and participant time and effort [19]. *Alive!* is immediately usable by organizations and businesses with little requirement for staff expertise and time commitment. Thus, *Alive!* provides the opportunity for widespread dietary and physical activity screening with immediate individualized feedback, which can then be followed by *Alive!*’s research-based effective intervention.

### Conclusions

In summary, these results show that participation in *Alive!* can result in significant improvements in important health parameters including physical and mental quality of life, self-assessed health status, self-efficacy for improving these health behaviors, and stage of adoption of change. Improvement in measures of presenteeism also suggests the possibility of economic benefits through improved productivity.

## Multimedia Appendix 1

Examples of *Alive!* assessment and feedback

## Multimedia Appendix 2

Examples of *Alive!* email and user’s home page

## Abbreviations

Alive!: a lifestyle intervention via email
CAPS: cross-cultural activity participation study
CDC: Centers for Disease Control and Prevention
FS: fats and sugars
FV: fruits and vegetables
HMO: health maintenance organization
HRAs: health risk assessments
KP: Kaiser Permanente of Northern California
NHANES: National Health and Nutrition Examination Survey
PA: physical activity
WIN: worksite internet nutrition
