# Supplementary material for: Development of Alive! (A Lifestyle Intervention Via Email), and Its Effect on Health-related Quality of Life, Presenteeism, and Other Behavioral Outcomes: Randomized Controlled Trial
Source: J Med Internet Res. 2008 Nov 19;10(4):e43. doi: 10.2196/jmir.1112 (PMC2629370; doi:10.2196/jmir.1112)
Supplement: Supplementary file 1 [file jmir_v10i4e43_app1.ppt]

## Slide 1
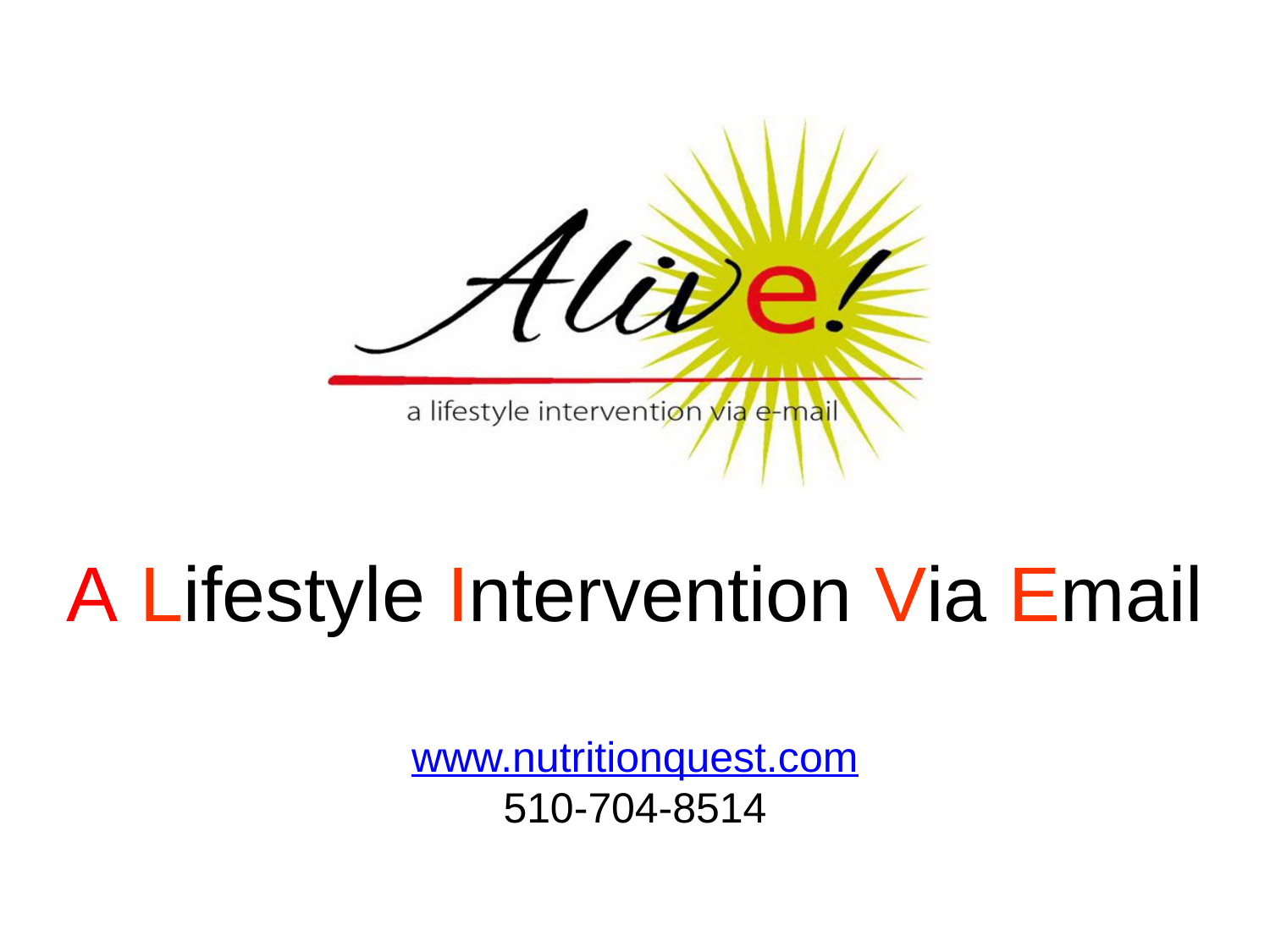

# A Lifestyle Intervention Via Email www.nutritionquest.com 510-704-8514

## Slide 2
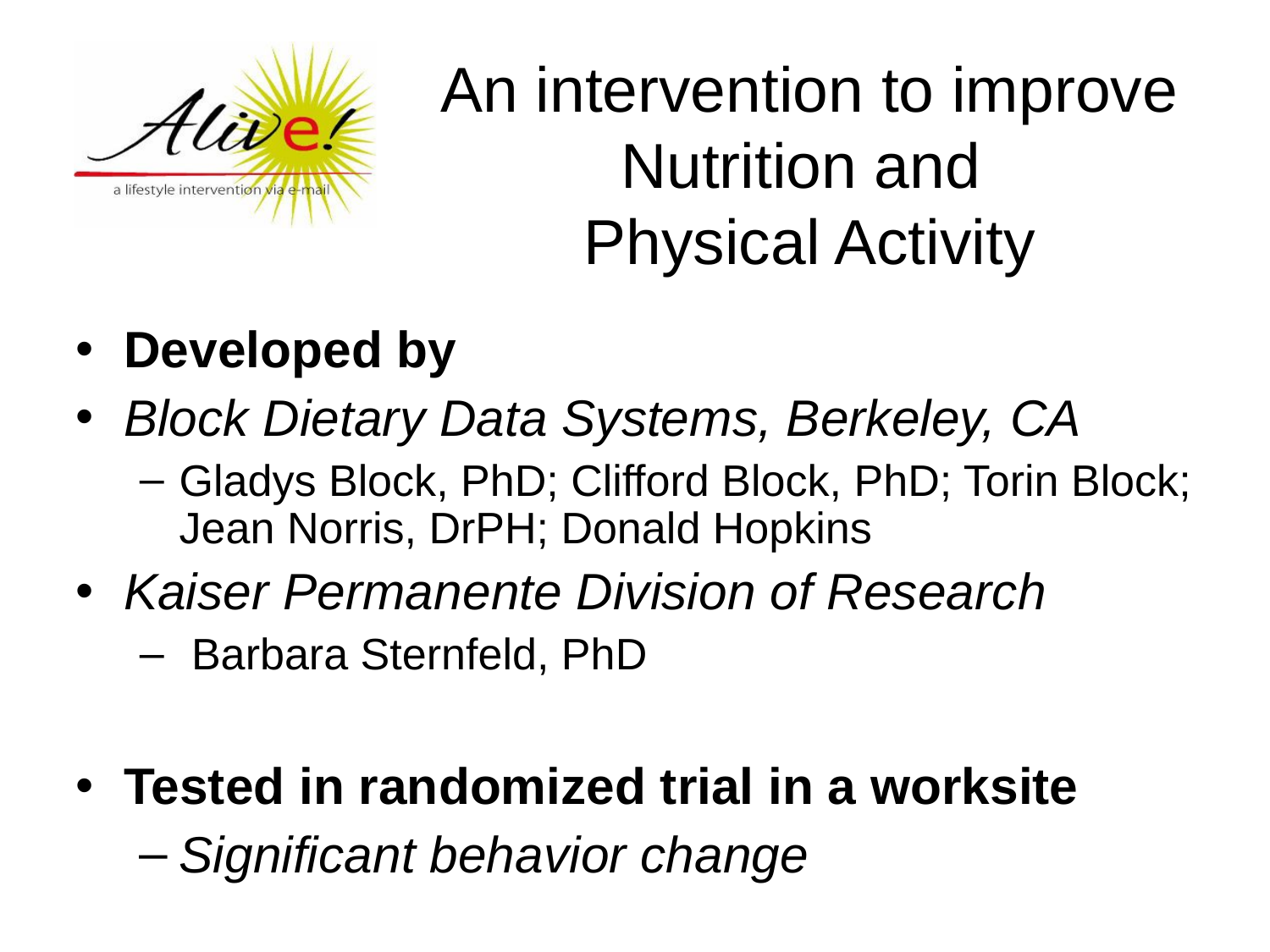

An intervention to improve Nutrition and
Physical Activity
# Developed by
Block Dietary Data Systems, Berkeley, CA
Gladys Block, PhD; Clifford Block, PhD; Torin Block; Jean Norris, DrPH; Donald Hopkins
Kaiser Permanente Division of Research
 Barbara Sternfeld, PhD
Tested in randomized trial in a worksite
Significant behavior change

## Slide 3
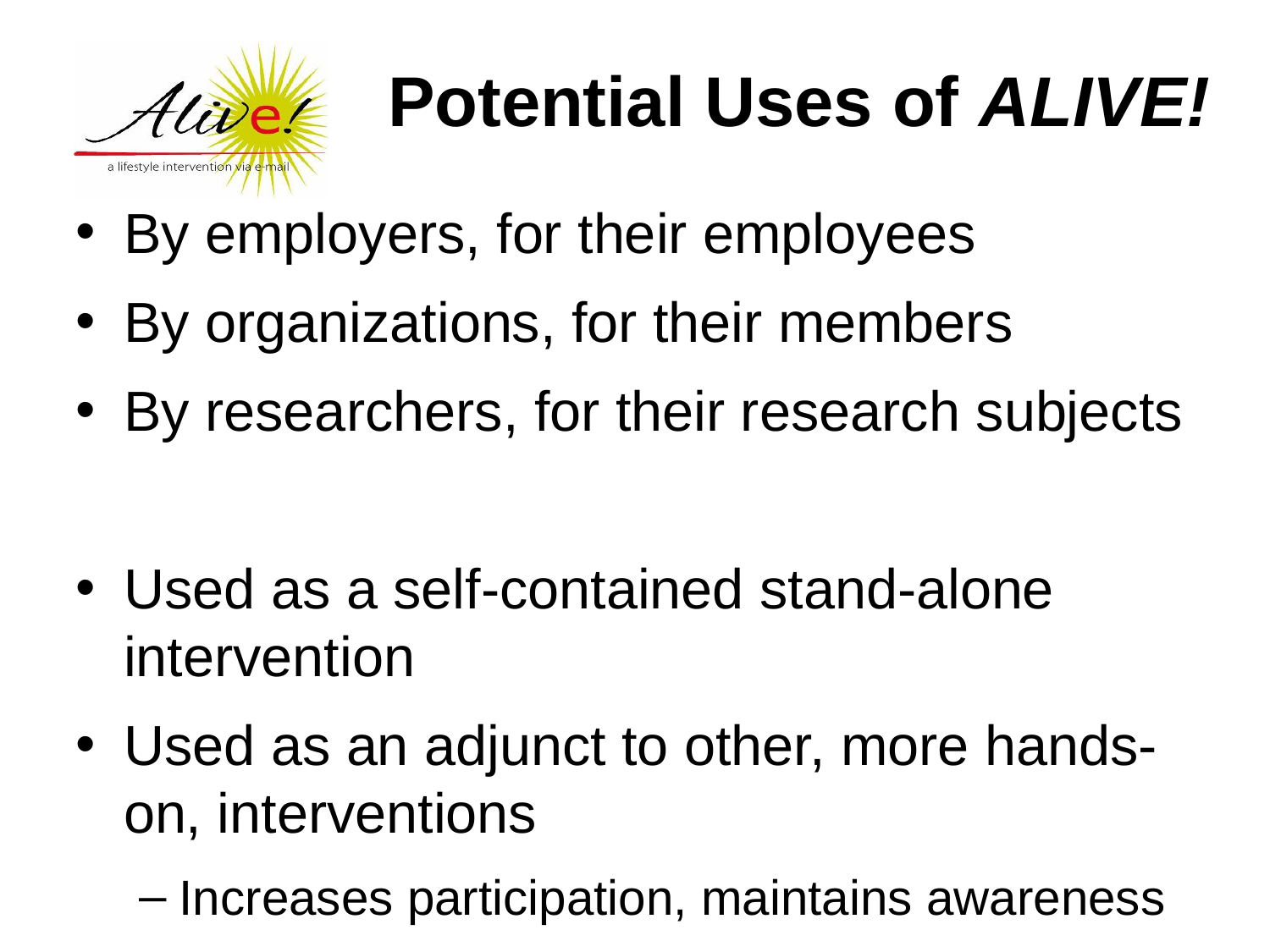

# Potential Uses of ALIVE!
By employers, for their employees
By organizations, for their members
By researchers, for their research subjects
Used as a self-contained stand-alone intervention
Used as an adjunct to other, more hands-on, interventions
Increases participation, maintains awareness

## Slide 4
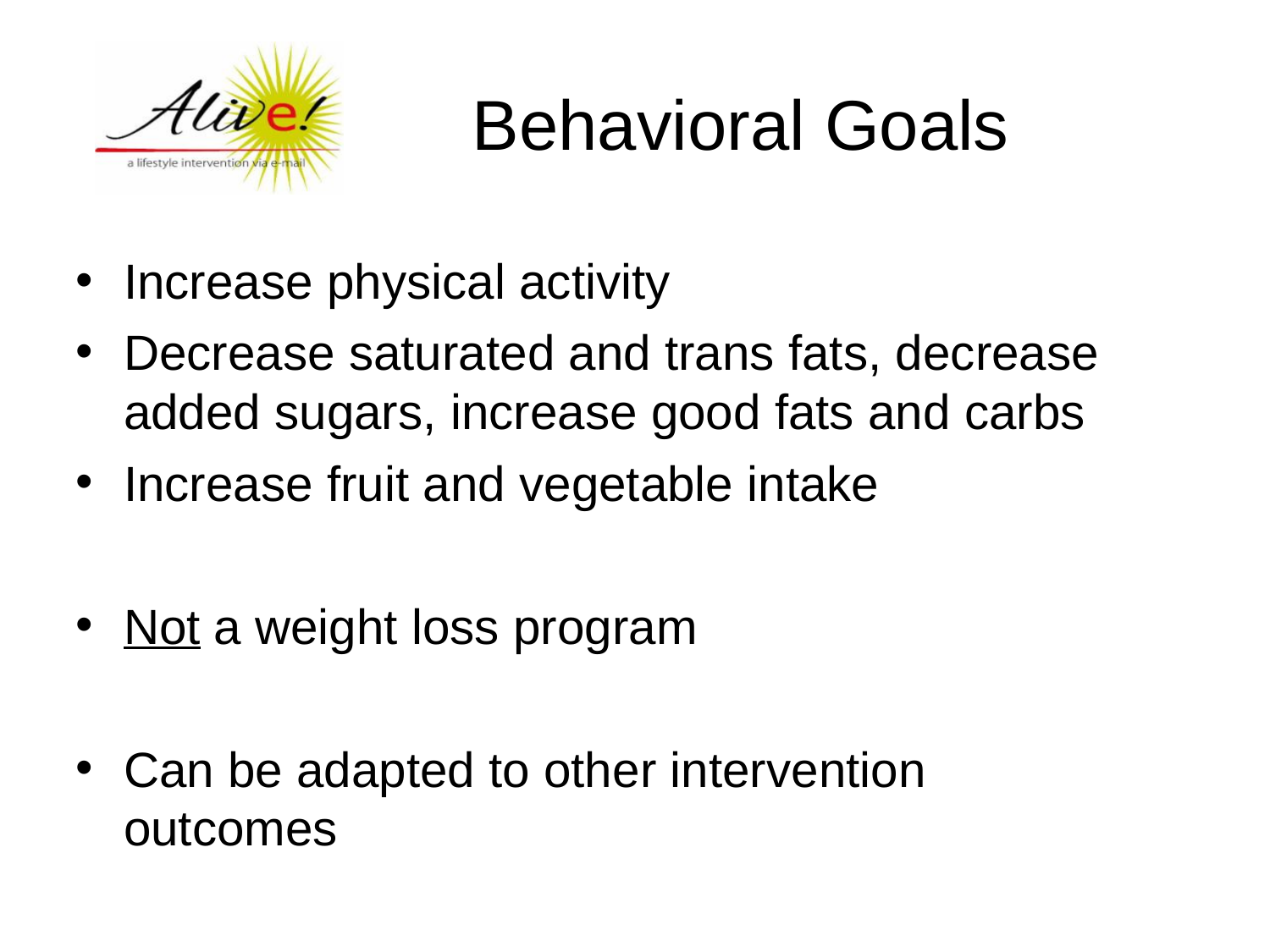

# Behavioral Goals
Increase physical activity
Decrease saturated and trans fats, decrease added sugars, increase good fats and carbs
Increase fruit and vegetable intake
Not a weight loss program
Can be adapted to other intervention outcomes

## Slide 5
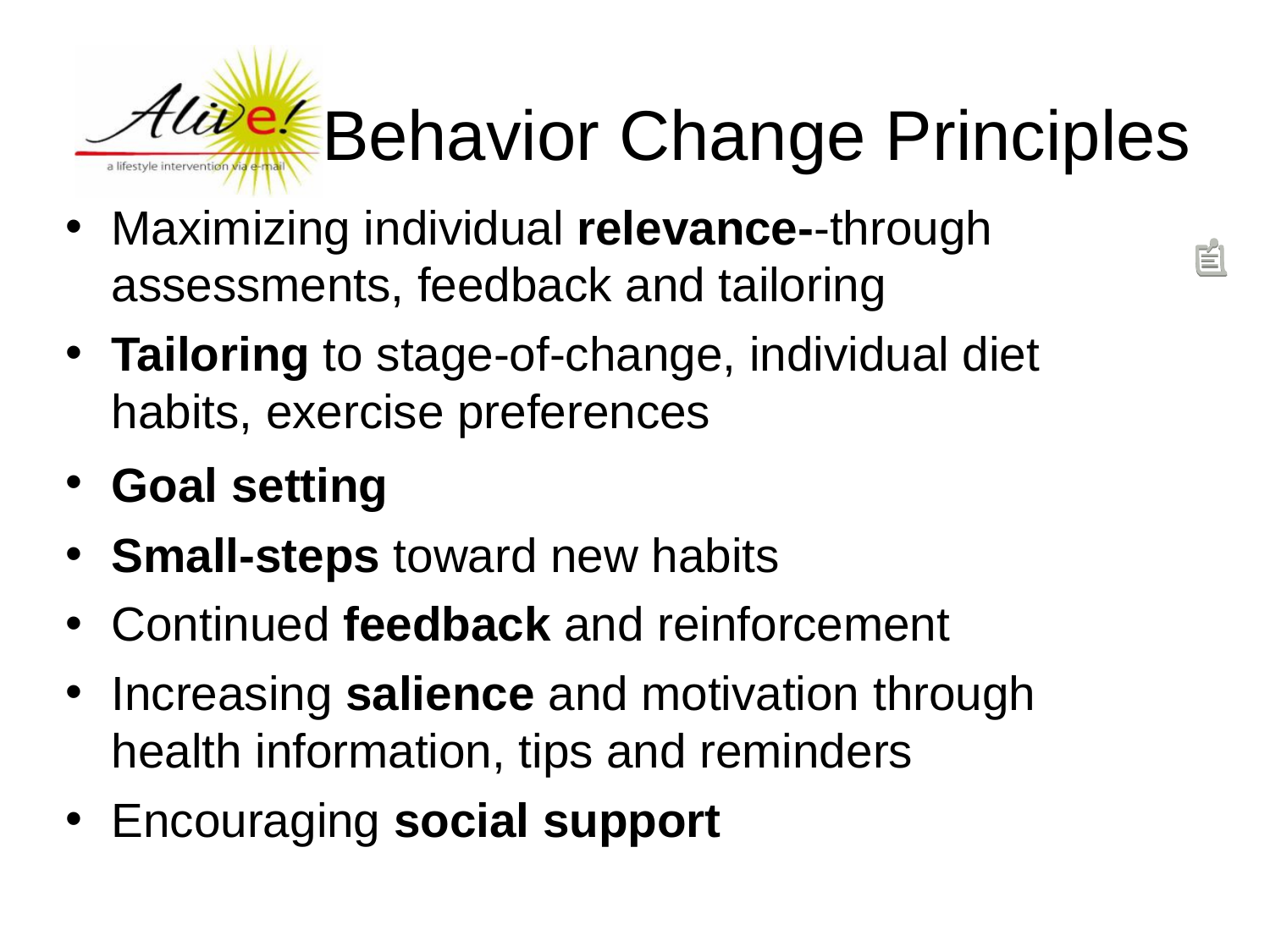

# Behavior Change Principles
Maximizing individual relevance--through assessments, feedback and tailoring
Tailoring to stage-of-change, individual diet habits, exercise preferences
Goal setting
Small-steps toward new habits
Continued feedback and reinforcement
Increasing salience and motivation through health information, tips and reminders
Encouraging social support

## Slide 6
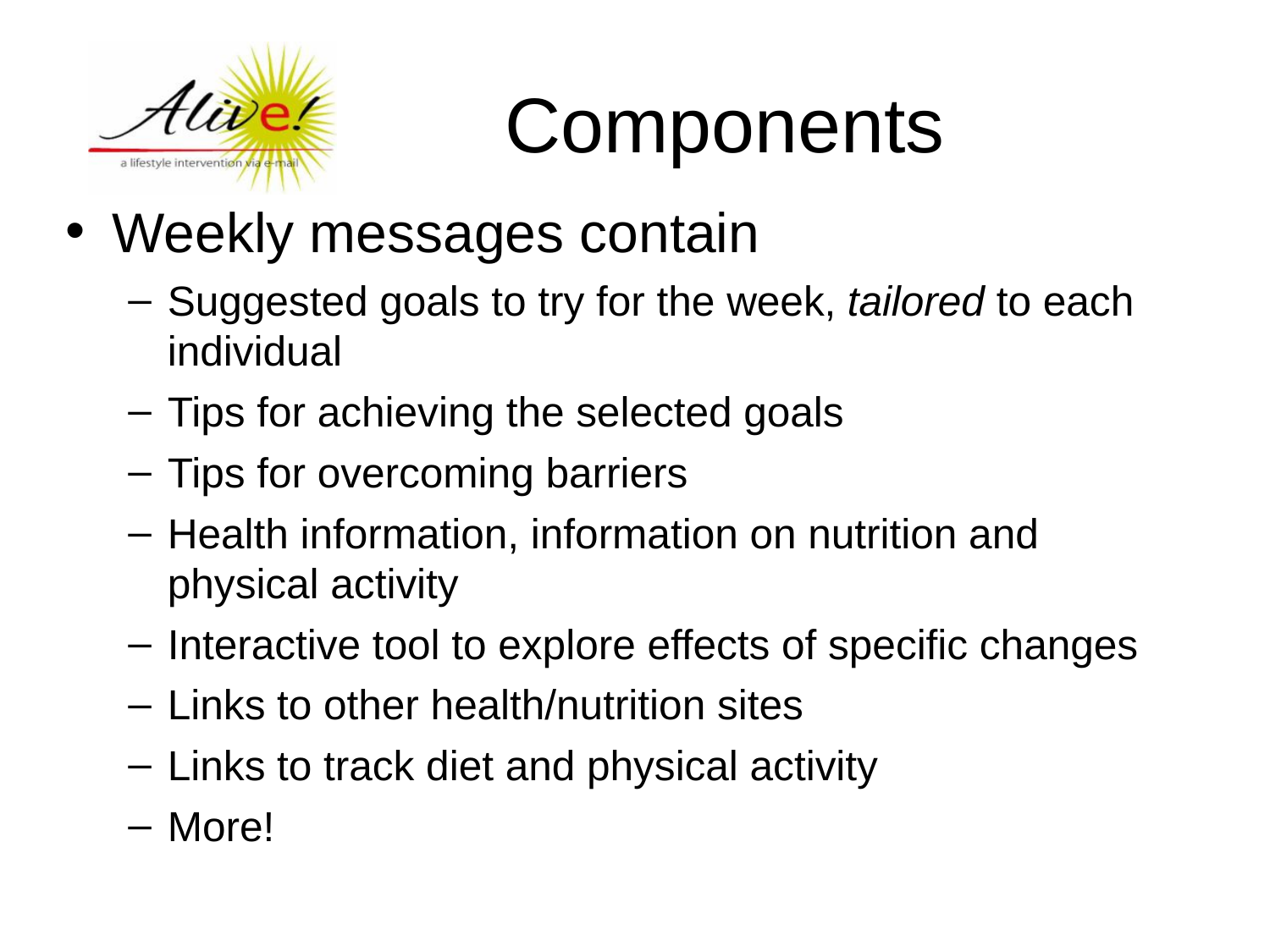

# Components
Weekly messages contain
Suggested goals to try for the week, tailored to each individual
Tips for achieving the selected goals
Tips for overcoming barriers
Health information, information on nutrition and physical activity
Interactive tool to explore effects of specific changes
Links to other health/nutrition sites
Links to track diet and physical activity
More!

## Slide 7
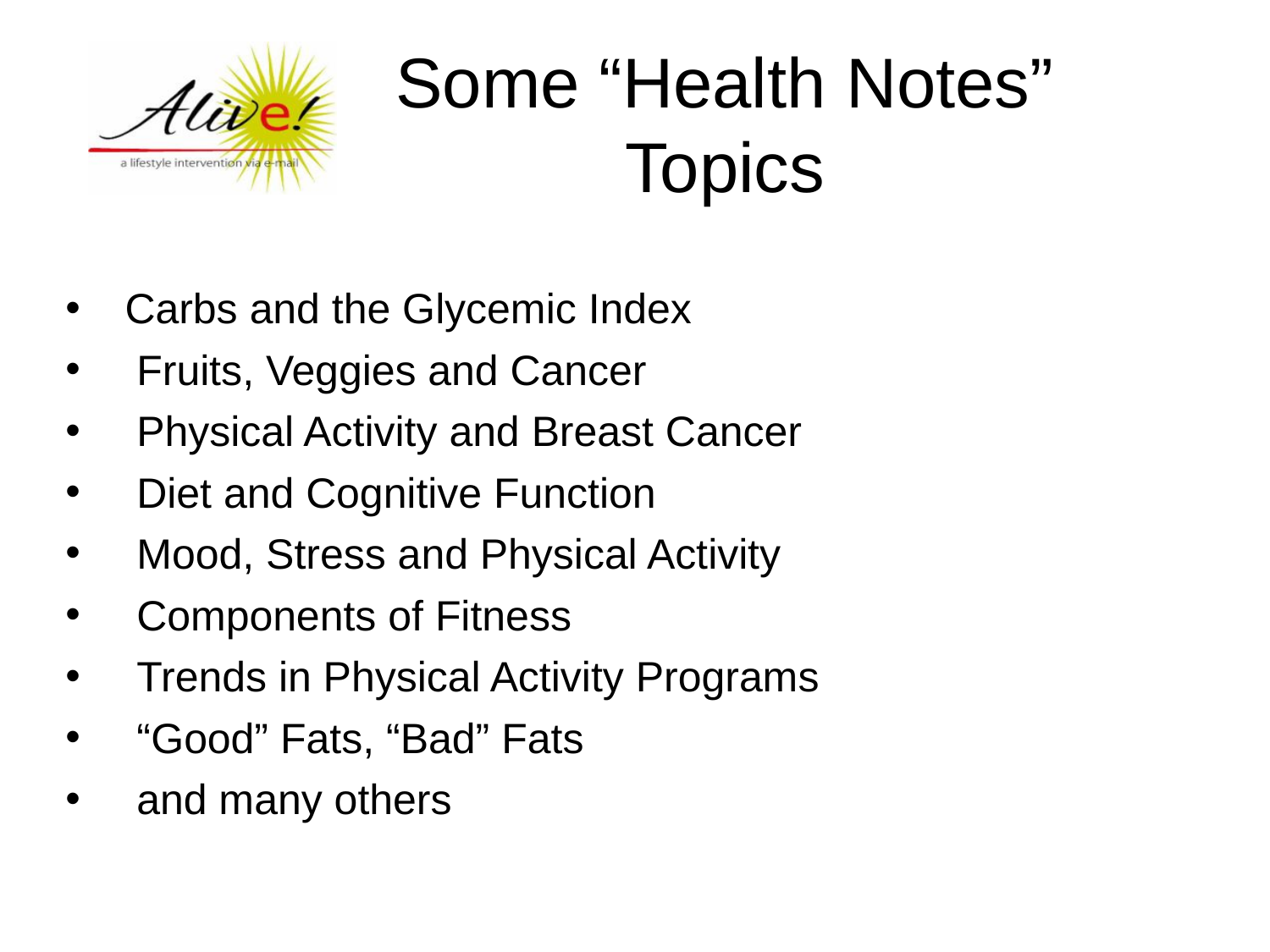

# Some “Health Notes” Topics
 Carbs and the Glycemic Index
 Fruits, Veggies and Cancer
 Physical Activity and Breast Cancer
 Diet and Cognitive Function
 Mood, Stress and Physical Activity
 Components of Fitness
 Trends in Physical Activity Programs
 “Good” Fats, “Bad” Fats
 and many others

## Slide 8
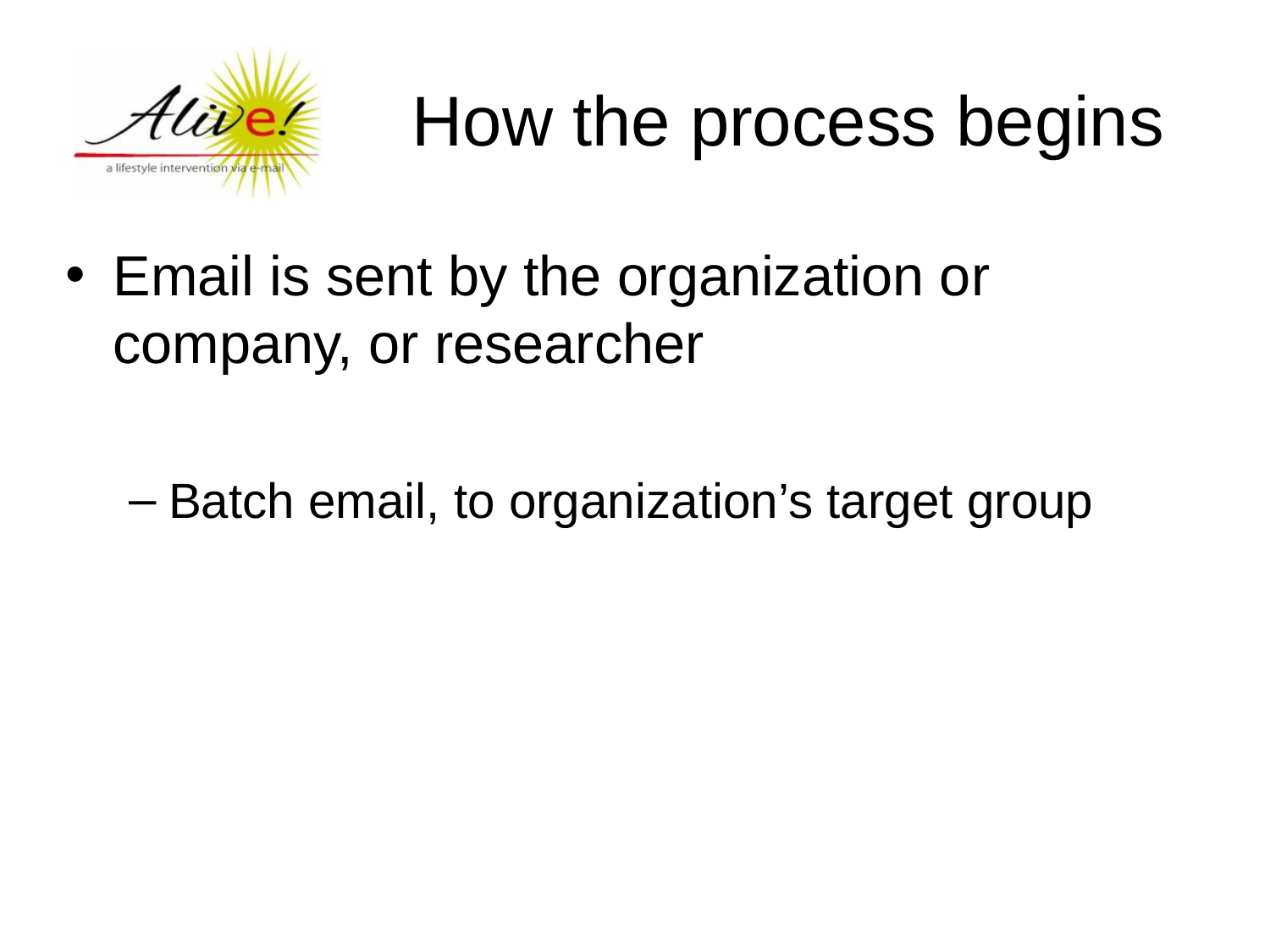

# How the process begins
Email is sent by the organization or company, or researcher
Batch email, to organization’s target group

## Slide 9
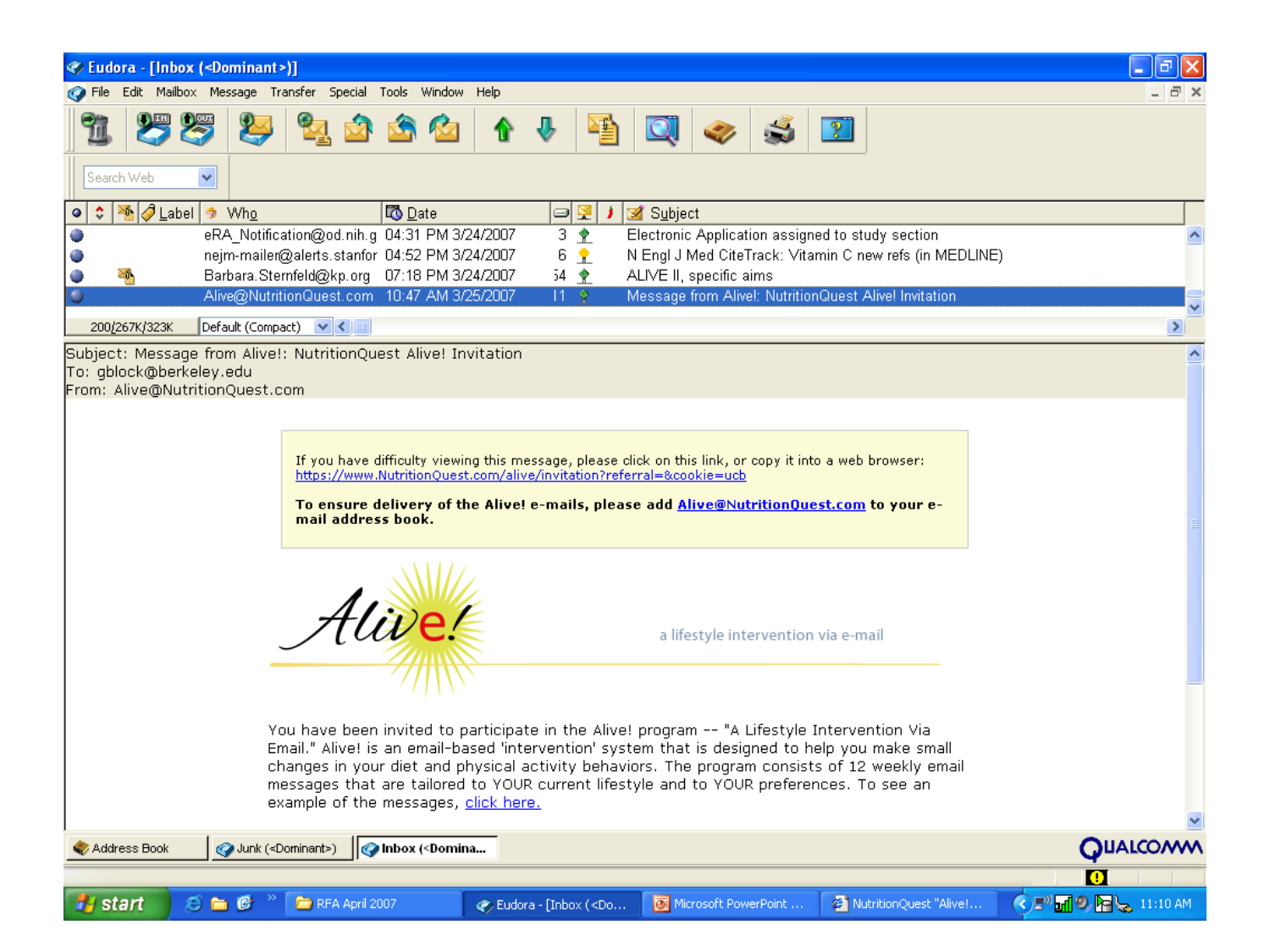

## Slide 10
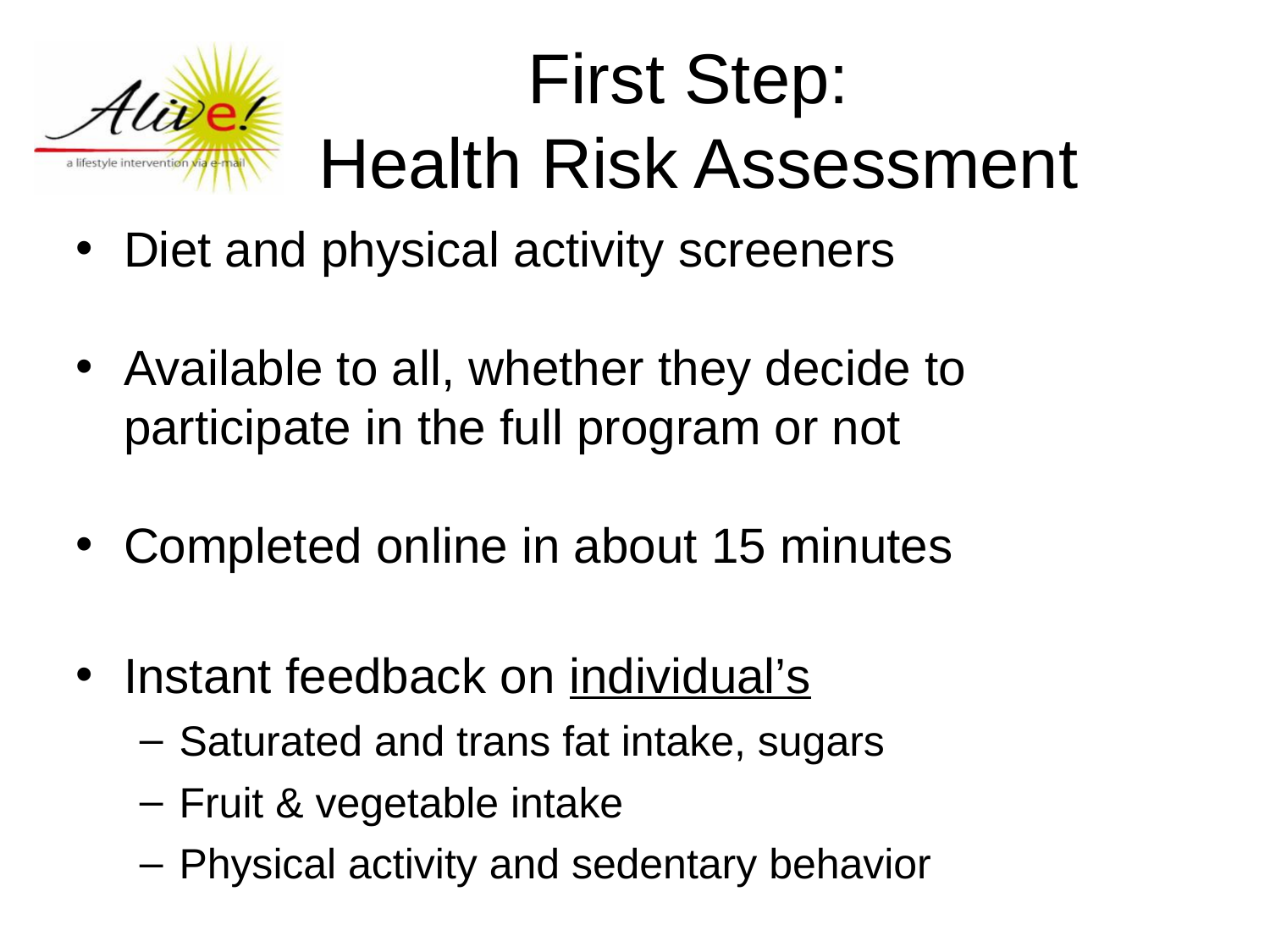

# First Step: Health Risk Assessment
Diet and physical activity screeners
Available to all, whether they decide to participate in the full program or not
Completed online in about 15 minutes
Instant feedback on individual’s
Saturated and trans fat intake, sugars
Fruit & vegetable intake
Physical activity and sedentary behavior

## Slide 11
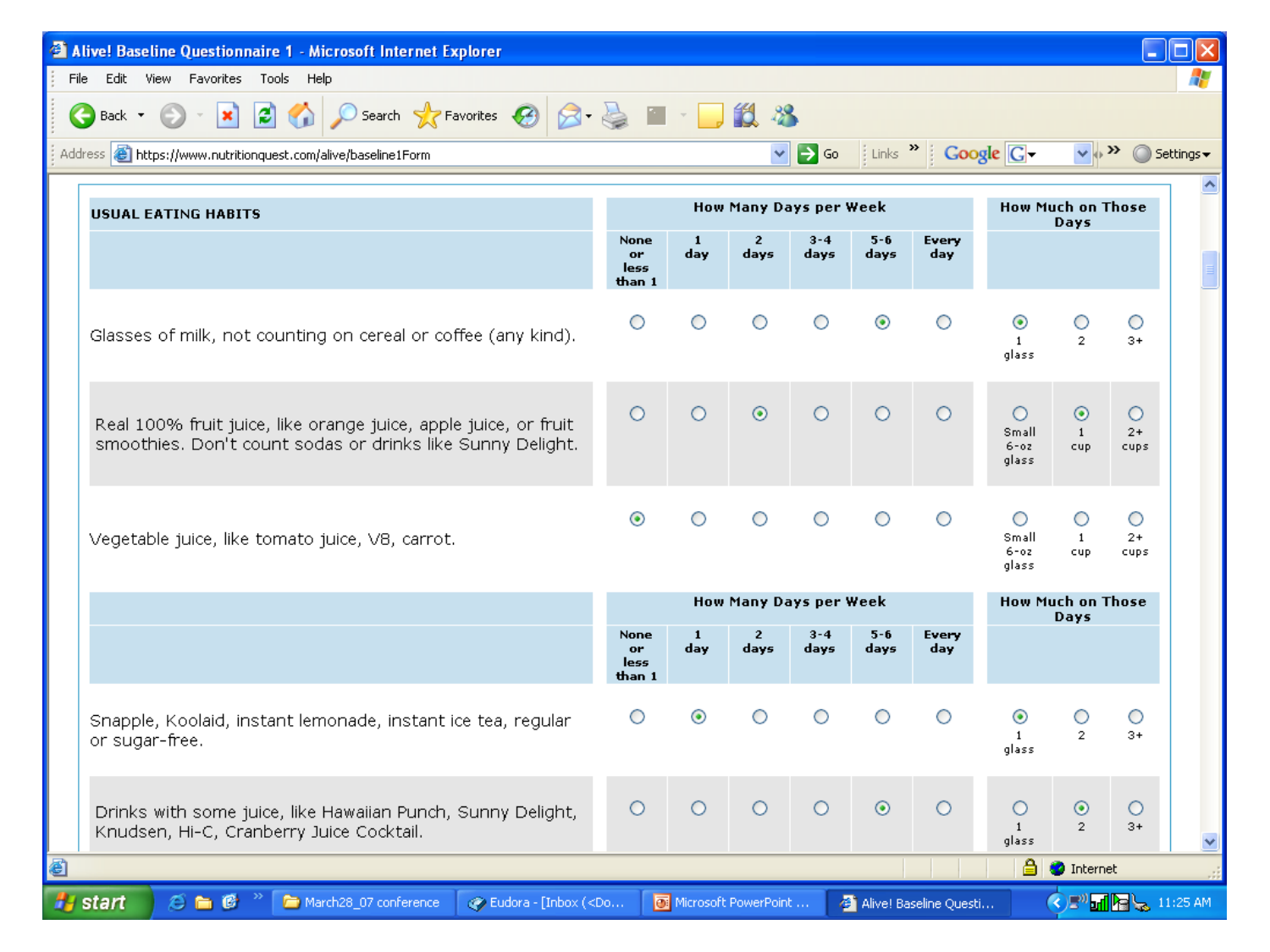

## Slide 12
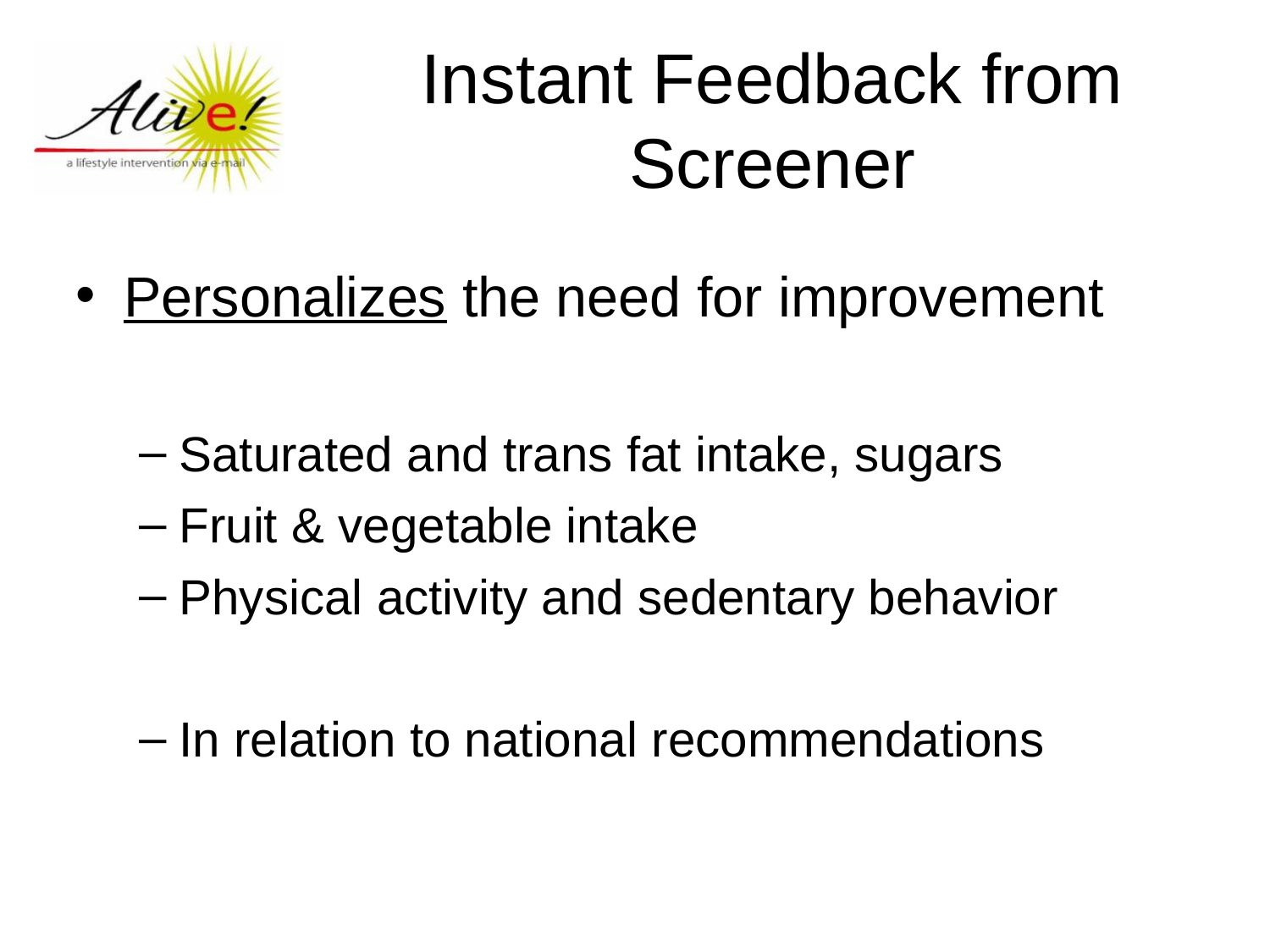

# Instant Feedback from Screener
Personalizes the need for improvement
Saturated and trans fat intake, sugars
Fruit & vegetable intake
Physical activity and sedentary behavior
In relation to national recommendations

## Slide 13
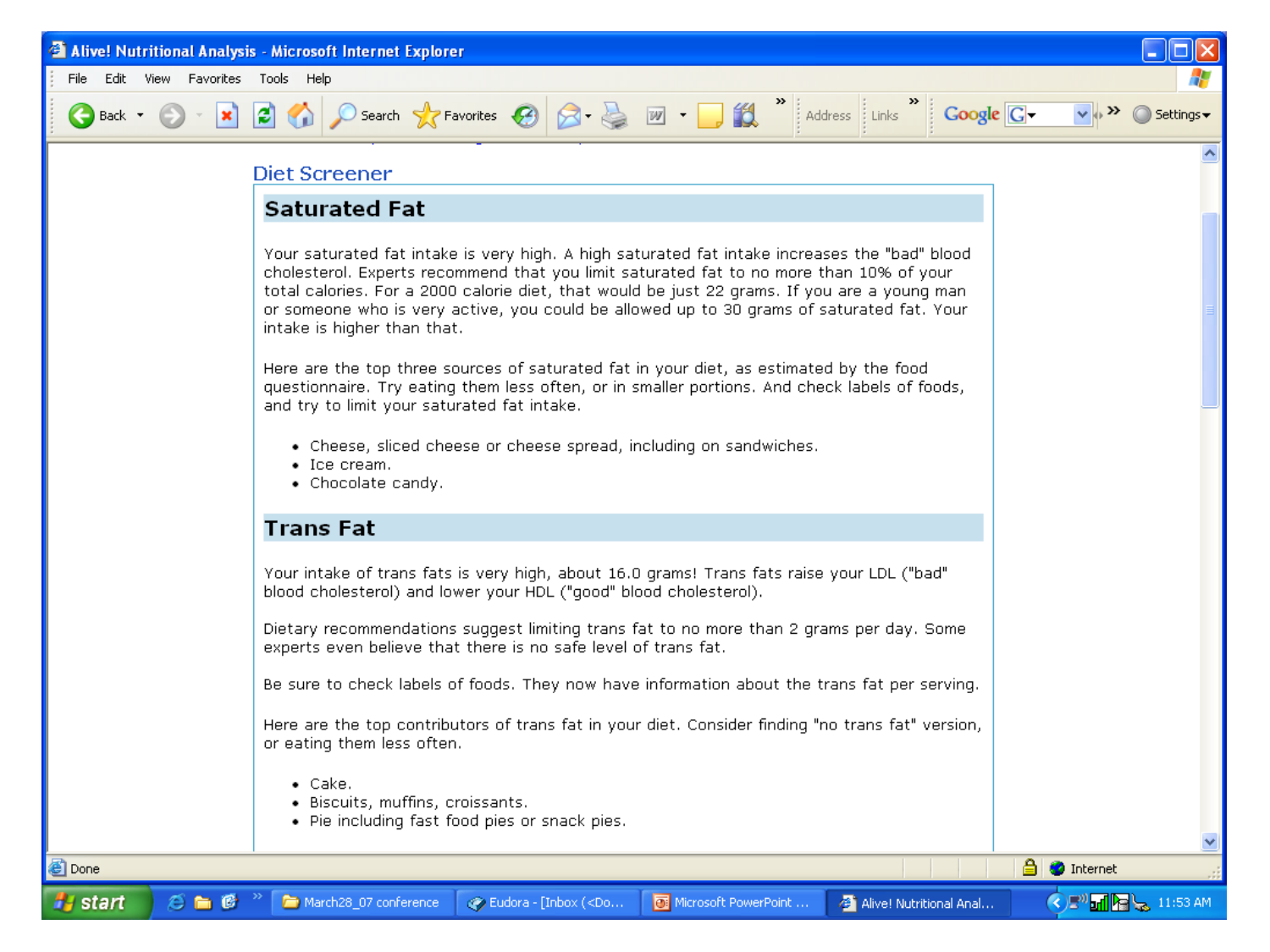

## Slide 14
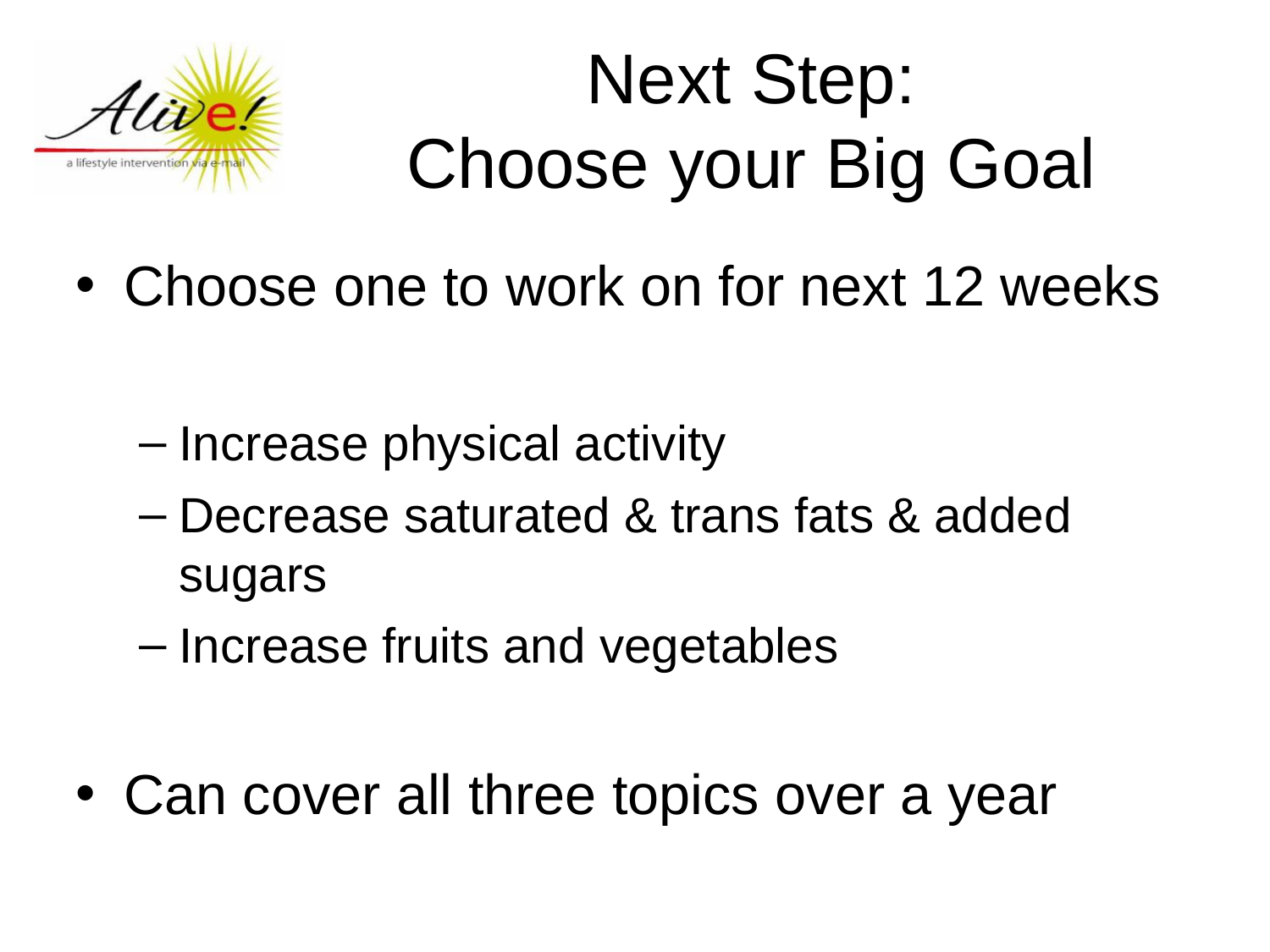

# Next Step: Choose your Big Goal
Choose one to work on for next 12 weeks
Increase physical activity
Decrease saturated & trans fats & added sugars
Increase fruits and vegetables
Can cover all three topics over a year

## Slide 15
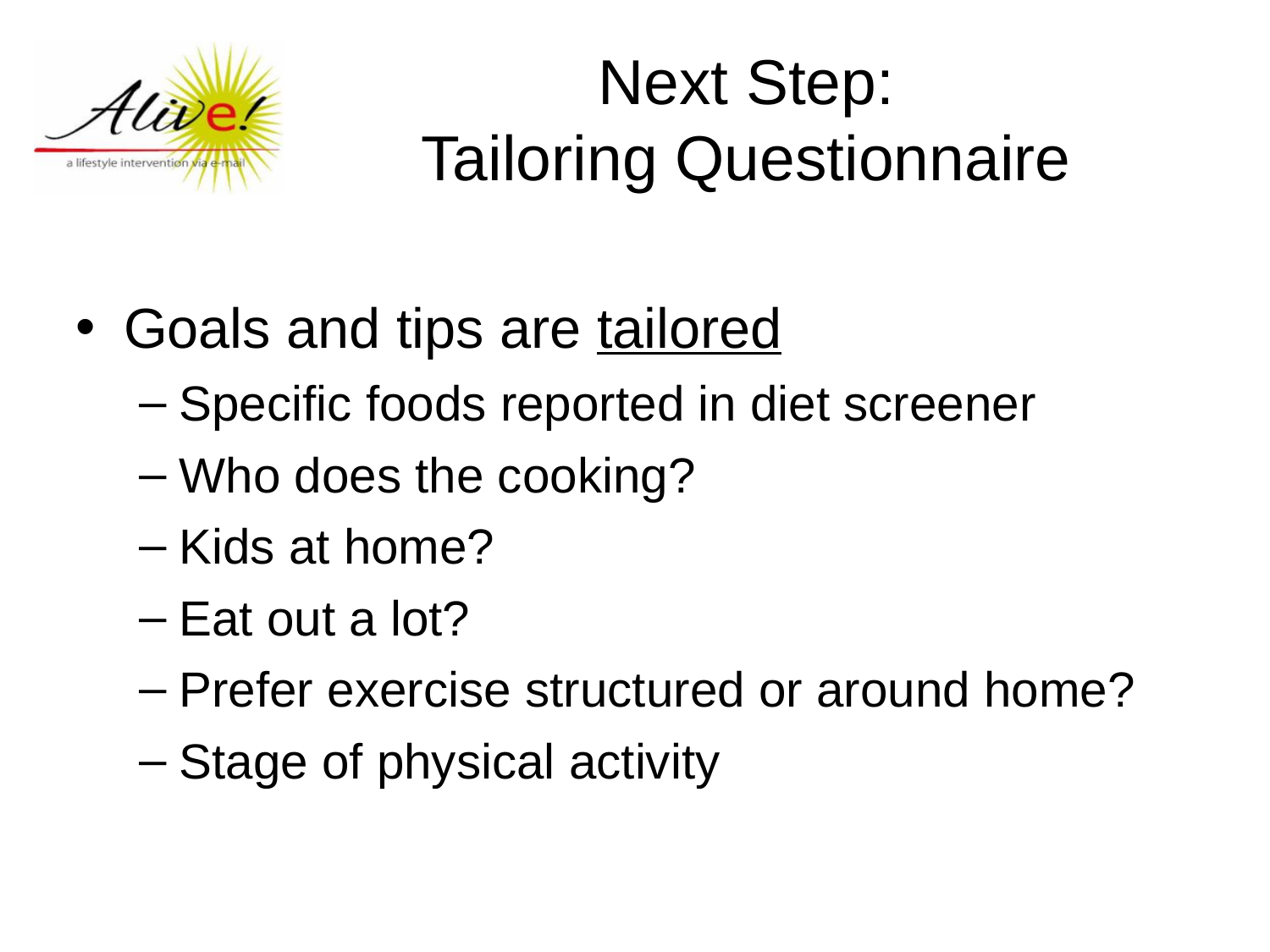

# Next Step: Tailoring Questionnaire
Goals and tips are tailored
Specific foods reported in diet screener
Who does the cooking?
Kids at home?
Eat out a lot?
Prefer exercise structured or around home?
Stage of physical activity

## Slide 16
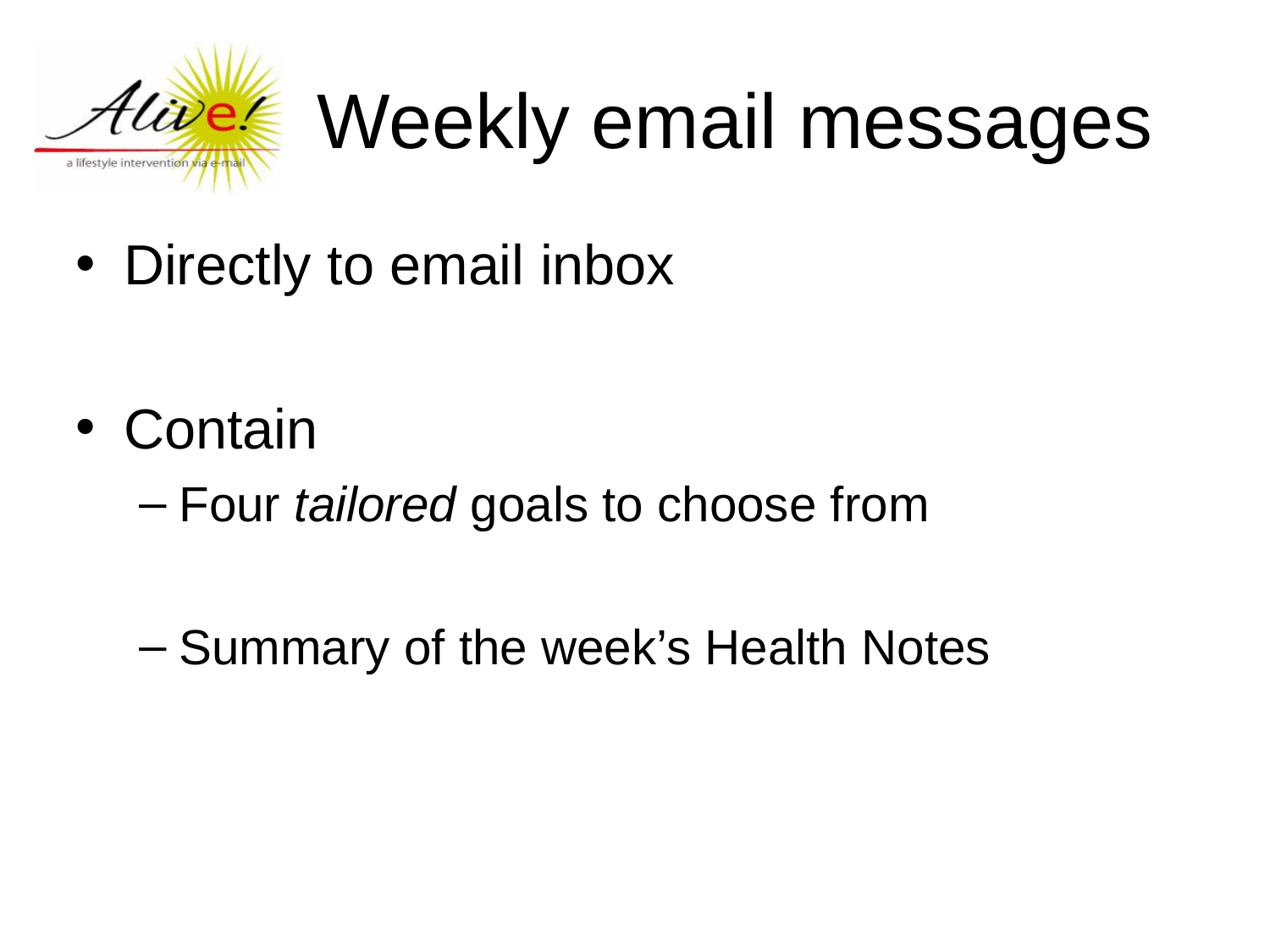

# Weekly email messages
Directly to email inbox
Contain
Four tailored goals to choose from
Summary of the week’s Health Notes

## Slide 17
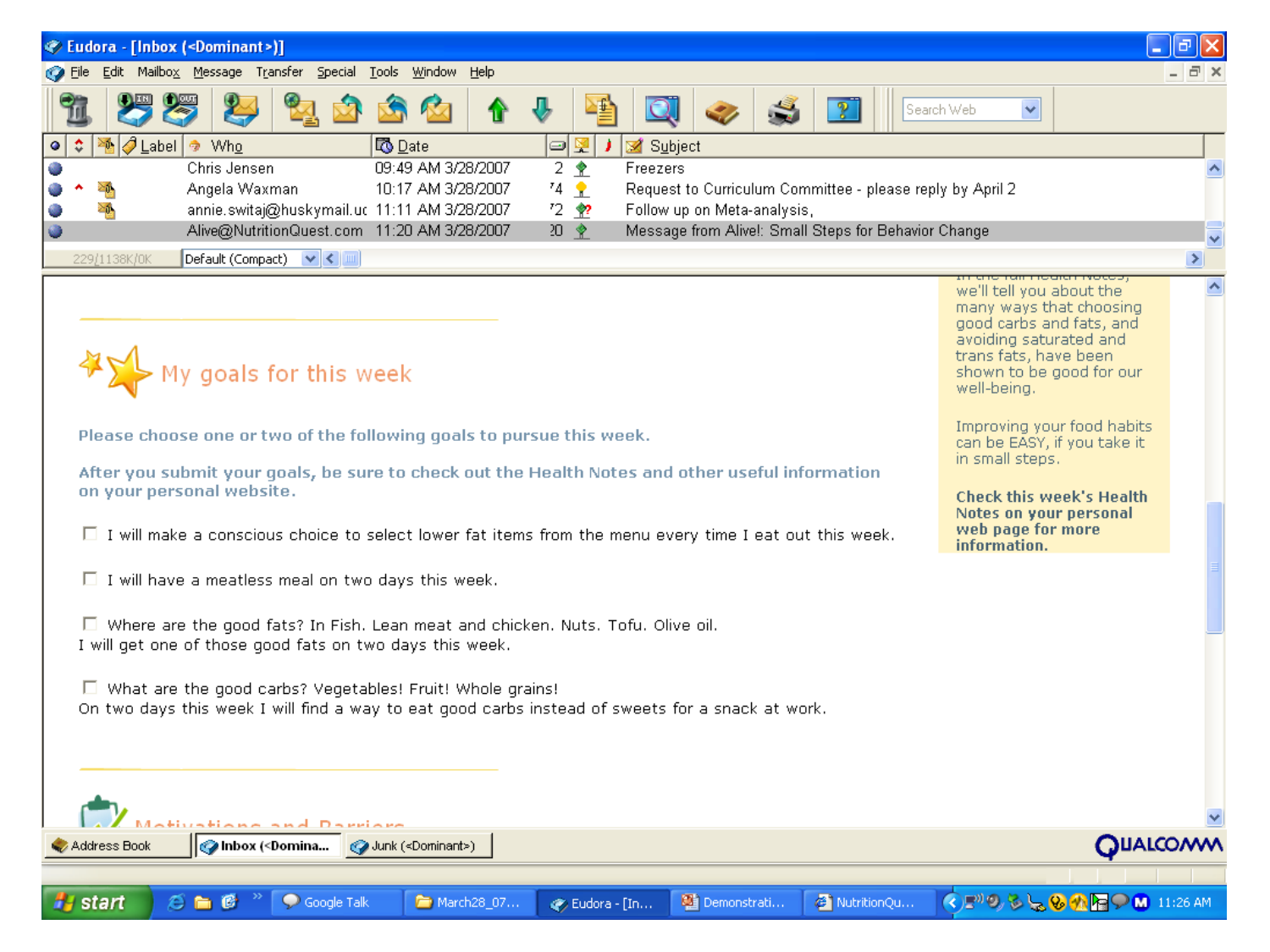

## Slide 18
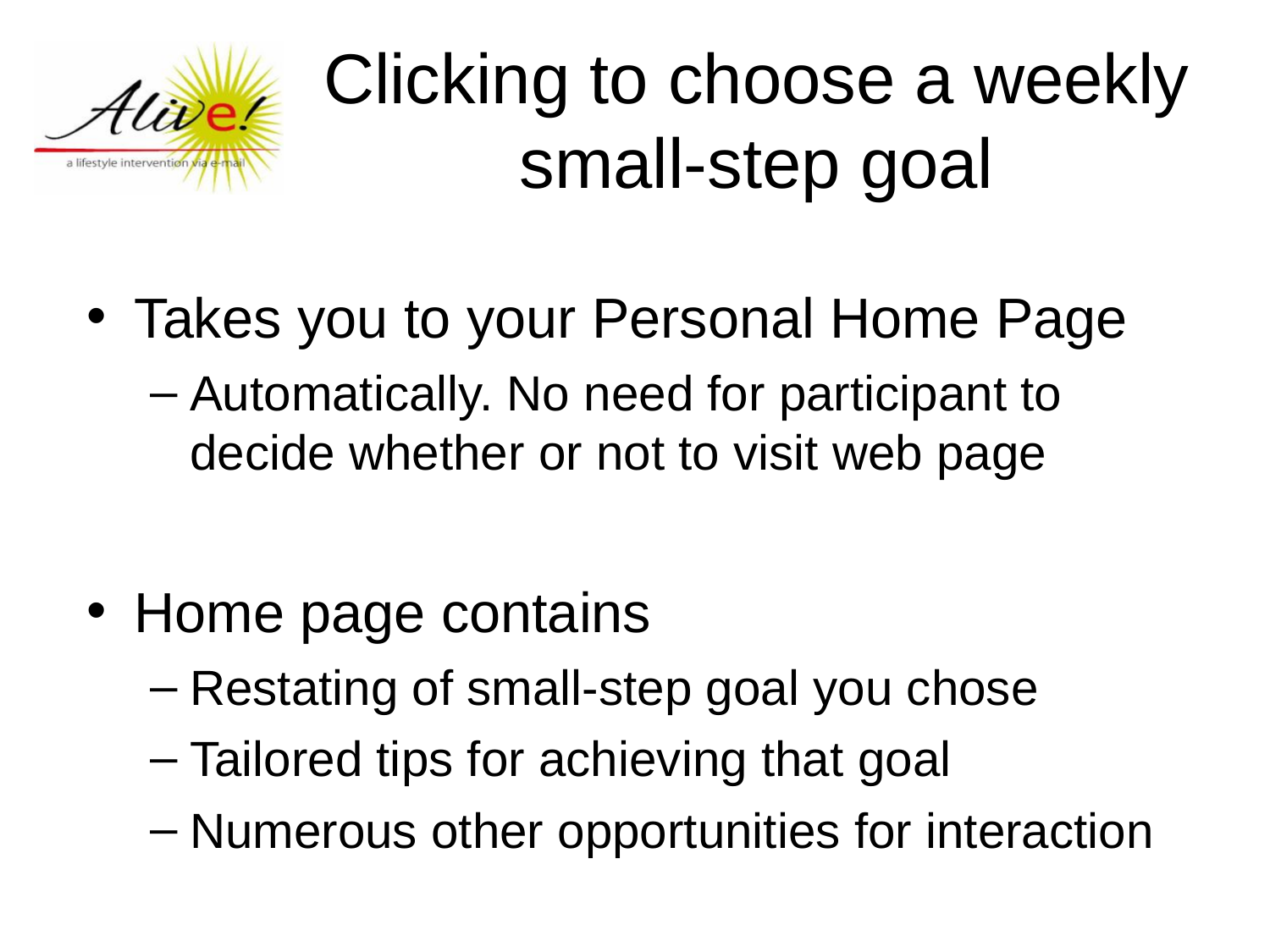

# Clicking to choose a weekly small-step goal
Takes you to your Personal Home Page
Automatically. No need for participant to decide whether or not to visit web page
Home page contains
Restating of small-step goal you chose
Tailored tips for achieving that goal
Numerous other opportunities for interaction

## Slide 19
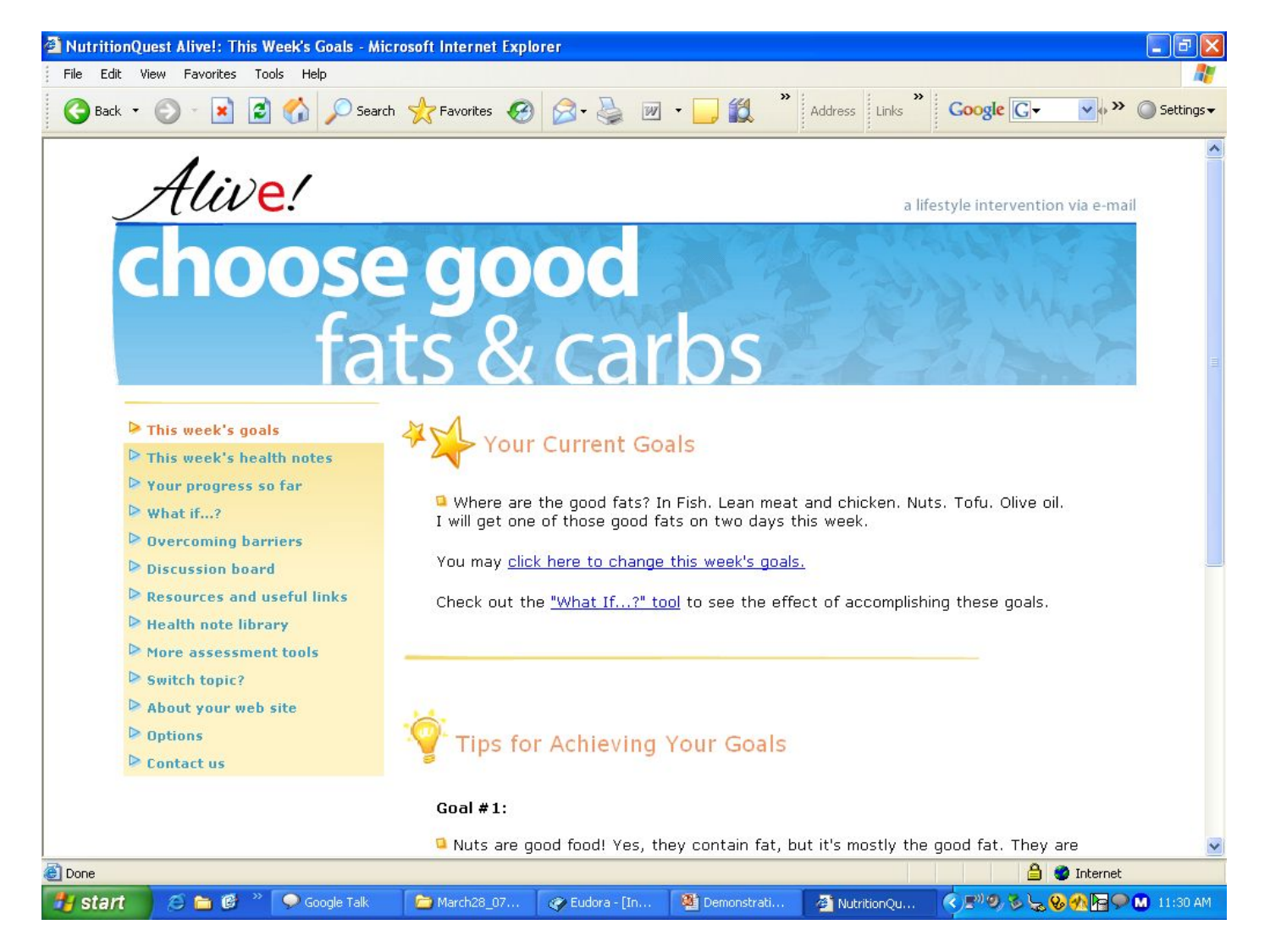

## Slide 20
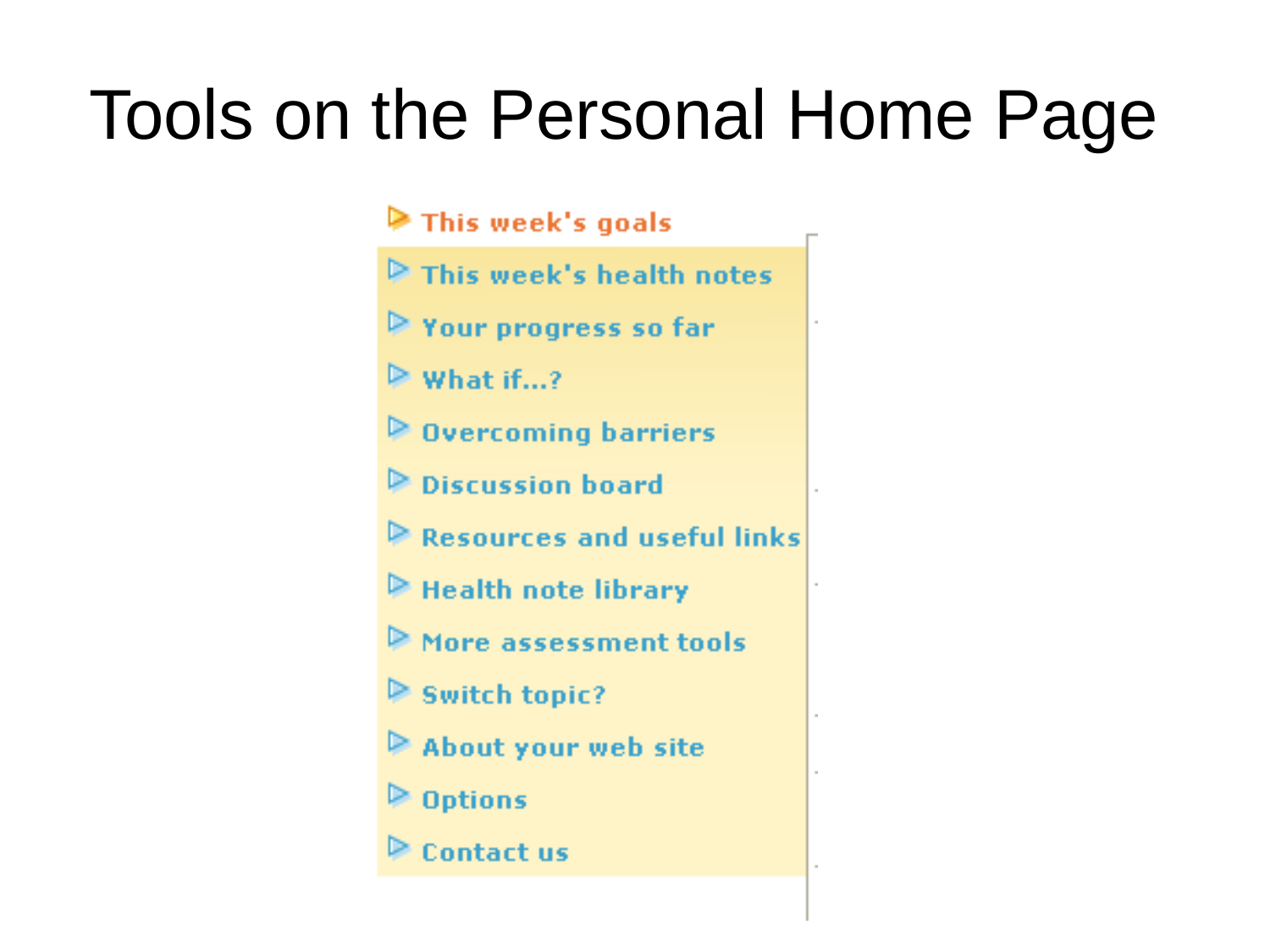

# Tools on the Personal Home Page

## Slide 21
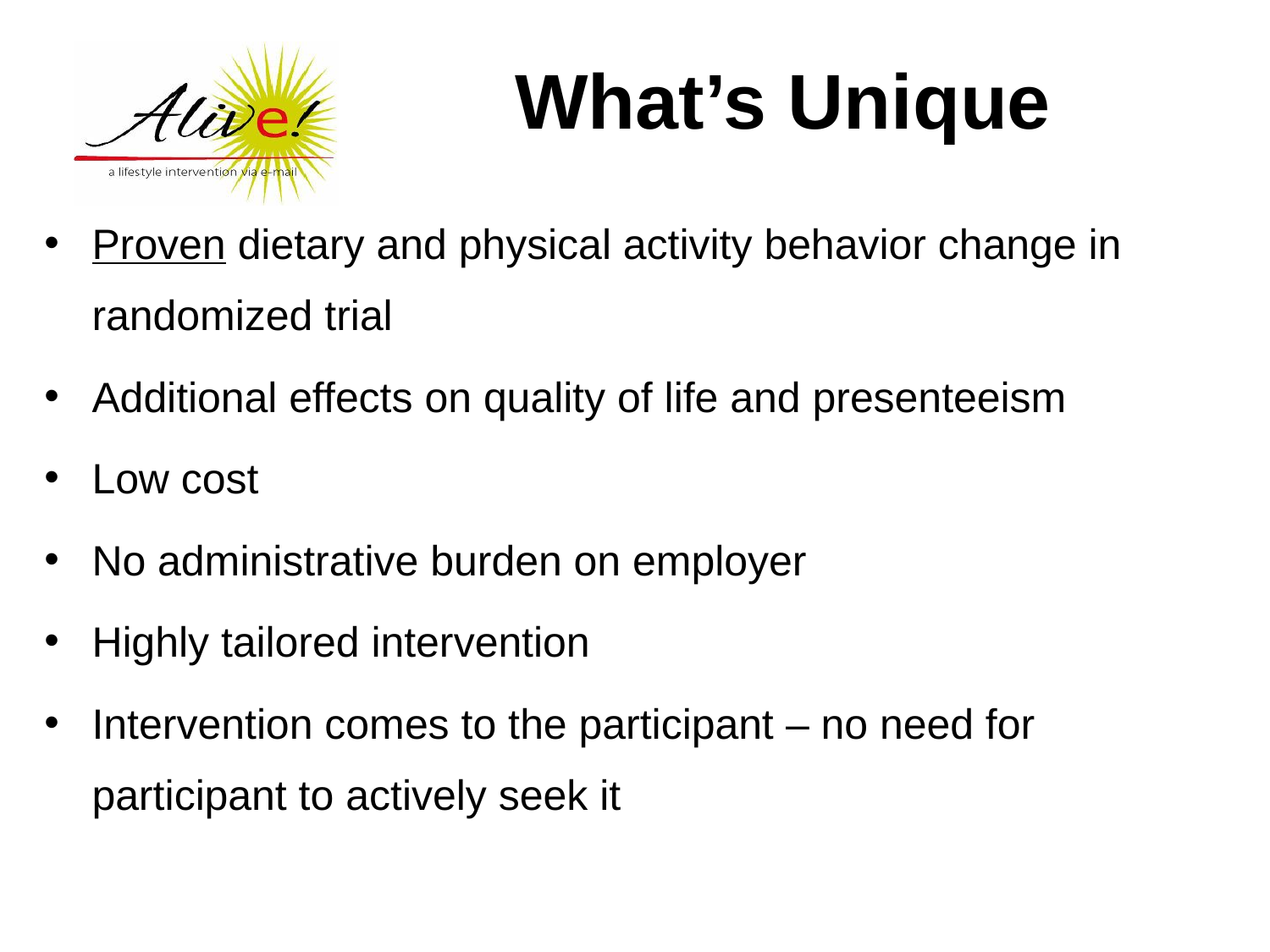

# What’s Unique
Proven dietary and physical activity behavior change in randomized trial
Additional effects on quality of life and presenteeism
Low cost
No administrative burden on employer
Highly tailored intervention
Intervention comes to the participant – no need for participant to actively seek it
