# Supplementary figures and images for: Development of Alive! (A Lifestyle Intervention Via Email), and Its Effect on Health-related Quality of Life, Presenteeism, and Other Behavioral Outcomes: Randomized Controlled Trial
Source: J Med Internet Res. 2008 Nov 19;10(4):e43. doi: 10.2196/jmir.1112 (PMC2629370; doi:10.2196/jmir.1112)

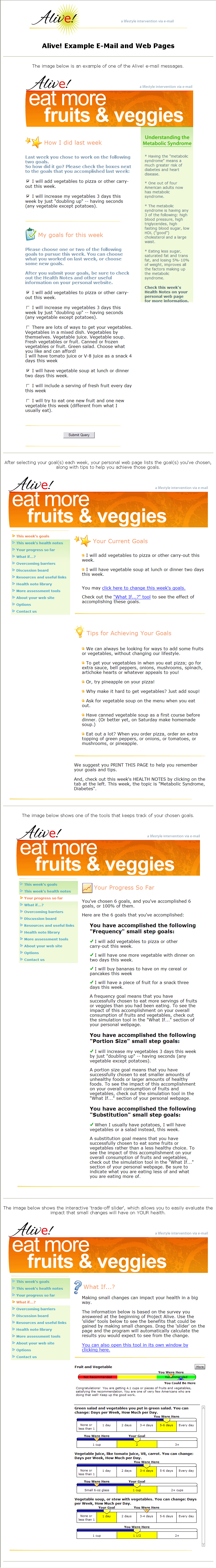

Supplement: Supplementary file 2 [file jmir_v10i4e43_app2.png]
